# Extracorporeal shock wave therapy for erectile dysfunction: rethinking study design, implementation, and analysis

**DOI:** 10.1093/bmb/ldaf004

**Published:** 2025-05-19

**Authors:** Janak Desai, Eric Huyghe, Gayle D Maffulli, Carmen Nussbaum-Krammer, Jessica Tittelmeier, Christoph Schmitz

**Affiliations:** Department of Urology, Samved Hospital, 100, Commerce Six Rd, Swastik Society, Navrangpura, Ahmedabad, Gujarat 380014, India; Department of Reproductive Medicine, Paule-de-Viguier Hospital, Toulouse University Hospital, 330 Av. de Grande Bretagne, 31300 Toulouse, France; Department of Urology, Andrology and Renal Transplantation, Rangueil Hospital, Toulouse University Hospital, 1 Av. du Professeur Jean Poulhès, 31400 Toulouse, France; UMR DEFE Inserm 1203, University of Toulouse 3, 2 Rue Charles Viguerie, 31300 Toulouse, France; SportsMed UK, Ealing, 29 Midhurst Road, London, W13 9XS, UK; Chair of Neuroanatomy, Institute of Anatomy, Faculty of Medicine, Ludwig-Maximilians-University of Munich (LMU Munich), Pettenkoferstr. 3, 80336 Munich, Germany; Chair of Neuroanatomy, Institute of Anatomy, Faculty of Medicine, Ludwig-Maximilians-University of Munich (LMU Munich), Pettenkoferstr. 3, 80336 Munich, Germany; Chair of Neuroanatomy, Institute of Anatomy, Faculty of Medicine, Ludwig-Maximilians-University of Munich (LMU Munich), Pettenkoferstr. 3, 80336 Munich, Germany

**Keywords:** acoustic pressure fields, clinical trials, extracorporeal shock wave therapy, ESWTerectile dysfunction, estimand, intercurrent events, missing data imputation

## Abstract

**Introduction:**

Extracorporeal shock wave therapy (ESWT) for erectile dysfunction (ED) presents a challenging paradox. While numerous clinical studies, systematic reviews, and meta-analyses have been published, indicating a substantial body of evidence supporting the efficacy and safety of ESWT, significant questions remain. Notably, the American Urological Association (AUA) continues to classify ESWT for ED as investigational (Evidence Level: Grade C), suggesting that the true therapeutic effect of ESWT may differ considerably from current estimates. This review aims to critically assess the evidence and propose strategies to address this unresolved discrepancy.

**Source of data:**

We systematically searched two electronic databases (PubMed and Ovid/Embase) and published systematic reviews on ESWT for ED and compiled a systematic literature review and meta-analysis based on 87 relevant studies.

**Areas of agreement:**

There is clear evidence that ESWT for ED is effective and can therefore be a valuable treatment modality in the management of ED.

**Areas of controversy:**

Current assessments of ESWT for ED as investigational by, e.g. the AUA may not stem from a lack of clinical studies, insufficient related basic science, or an inadequate number of systematic reviews and meta-analyses. Instead, the deficits lie in the area of the scientific quality of the clinical studies published to date.

**Growing points:**

We hypothesize that this unfortunate situation will only change if the following aspects are rigorously considered in future clinical studies on ESWT for ED: adequate characterization and reporting of extracorporeal shock waves, appropriate handling of missing data and intercurrent events, and comprehensive classification of ESWT in the overall context of the available treatment options for ED.

**Areas timely for developing research:**

We are convinced that the consistent implementation of these aspects will significantly contribute to establishing ESWT as the first truly regenerative therapy in the management of ED. This overall aim justifies the corresponding efforts for the benefit of our patients.

## Introduction

Erectile dysfunction (ED) is a prevalent condition, primarily affecting men over 40 years of age, and its incidence continues to rise globally.^1–3^ ED is characterized by the persistent or recurrent inability to achieve or maintain an erection sufficient for satisfactory sexual performance. The pathophysiology of ED is multifactorial, involving a complex interplay of organic, psychogenic, and mixed factors.^3^ Common comorbidities associated with ED include cardiovascular disease, diabetes, and neurological disorders, as well as post-radical prostatectomy conditions.^2–4^ If left untreated, ED can significantly impact a patient's quality of life, leading to reduced self-esteem, strained interpersonal relationships, and increased risks of anxiety and depression.^2^^,^^3^

Current treatment options for ED, as recommended by guidelines from the European Urological Association and the American Urological Association (AUA), include phosphodiesterase type 5 inhibitors (PDE5i), inflatable penile prostheses, vacuum erection devices, intracavernosal injections of alprostadil and other drugs, and intraurethral administration of alprostadil.^5–7^ However, none of these therapies provide curative or regenerative solutions for ED.

In recent years, extracorporeal shock wave therapy (ESWT) has emerged as a promising, non-invasive treatment for ED.^3^^,^^7^^,^^8^ Initially developed for lithotripsy to break kidney stones,^9^ ESWT has been successfully applied for over 30 years in treating various musculoskeletal disorders.^10–12^ The molecular and cellular mechanisms of ESWT have been well studied, revealing their beneficial effects on bone and cartilage as well as on connective tissue, muscle tissue, and nerve tissue.^13^ Of particular interest is the ability of ESWT to induce functional angiogenesis,^13–15^ making it an appealing option for addressing ED, which often involves impaired blood flow.

Despite its potential, the clinical efficacy of ESWT for ED remains controversial. Systematic reviews and meta-analyses published between 2017 and 2024 (summarized in Table 1) suggest that ESWT is an effective intervention for ED.^16–31^ The EAU recommends ESWT, either alone or in combination with PDE5i, for patients with mild vasculogenic ED, for those who prefer alternatives to oral therapy, and for poor responders to PDE5i.^32^ The European Society for Sexual Medicine also supports ESWT as a treatment option.^5^ In contrast, the most recent AUA guidelines classify ESWT as an investigational treatment (Conditional Recommendation, Evidence Level: Grade C),^6^ indicating low to very low confidence in the treatment’s effect.^33^

**Table 1:** Conclusions regarding the efficacy of ESWT for ED in systematic reviews and meta-analyses published between 2017 and 2024

**R**	**NFA**	**Y-P**	**Conclusion**
^16^	Hinojosa-Gonzalez	2024	Our network meta-analysis suggests that low-intensity extracorporeal shockwave therapy is an effective intervention for ED, as measured by increases in the IIEF-EF.
^17^	Vieiralves	2023	The literature presents little scientific evidence but suggests good results with the use of LI-EST for ED.
^18^	Mason	2023	The examined studies present encouraging results for the use of LI-ESWT to treat diabetic men with ED.
^19^	Liu	2022	Compared with placebo treatment, LI-ESWT alleviates ED symptoms in patients, particularly those who have mild or moderate ED.
^20^	Rho	2022	The results of this analysis indicate that LI-ESWT showed a statistically significant effect on early recovery in penile rehabilitation of ED following RP.
^21^	Yao	2022	The results of this meta-analysis suggest that treatment plans with an energy density of 0.09 mJ/mm^2^ and pulses number of 1500 to 2000 are more beneficial to IIEF in ED patients. In addition, IIEF improvement was more pronounced in patients with moderate ED after extracorporeal shockwave therapy.
^22^	Canguven	2021	The present review found that LI-ESWT has a role in ED treatment in laboratory studies, but its role in human clinical trials is still controversial.
^23^	Kałka	2021	Evidence exists that LI-ESWT generated with an EH unit is effective.
^24^	Ochoa	2021	SW may have a theoretical impact on the vascular etiology of organic ED.
^25^	Dong	2019	In meta-analysis of seven RCTs with men who received LI-ESWT for ED, there was evidence that the IIEF-EF and EHS experienced improvements following LI-ESWT.
^26^	Sokolakis	2019	The present meta-analysis provided results, showing that LI-ESWT significantly improves erectile function in patients with vasculogenic ED.
^27^	Campbell	2019	This therapeutic strategy appears to be well tolerated with short-term benefits.
^28^	Man	2018	These studies suggest that LI-ESWT could significantly improve the IIEF and EHS of patients with ED.
^29^	Angulo	2017	According to the literature, treatment with LI-ESWT for ED is effective, both in the short and medium term. LI-ESWT has been described as more effective than placebo in the short term.
^30^	Lu	2017	The number of studies of LI-ESWT for ED has increased dramatically in recent years. Most of these studies presented encouraging results, regardless of variation in LI-ESWT setup parameters or treatment protocols. These studies suggest that LI-ESWT could significantly improve the IIEF and EHS of ED patients.
^31^	Clavijo	2017	In this meta-analysis of seven RCTs, treatment of ED with LI-ESWT resulted in a significant increase in IIEF-EF scores.

ED, erectile dysfunction; EHS, erection hardness score; IIEF, International Index of Erectile Function; IIEF-EF, IIEF—Erectile Domain; N-FA, name of the first author; R, reference; RCTs, randomized controlled trials; RP, radical prostatectomy; SW, shock waves; Y-P, year of publication.

A critical review of the available literature reveals several gaps in existing systematic reviews and meta-analyses, including the inadequate characterization of shock wave parameters (such as energy density and the type of extracorporeal shock waves (ESWs) used), inconsistent handling of missing data and intercurrent events, and a lack of clarity regarding the integration of ESWT with other ED treatment modalities. These unresolved issues warrant further investigation to optimize clinical protocols.

In this systematic review, we evaluate clinical studies on ESWT for ED published up to 27 September 2024, with a focus on the aforementioned critical aspects. Our analysis of 87 studies suggests that the current evidence is insufficient to fully support ESWT as a standardized treatment for ED. Based on our findings, we call for a reevaluation of study designs and methodologies in future clinical trials, with an emphasis on addressing the key gaps identified in the literature.

## Materials and methods

**Figure 1:**
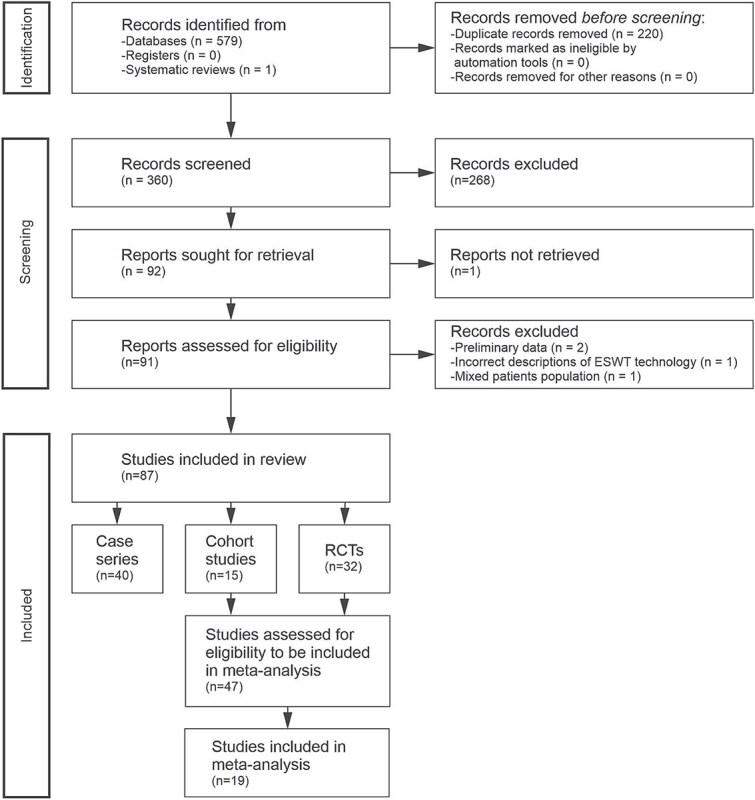
Systematic review flowchart of the literature search regarding ESWT for ED, performed according to the PRISMA guidelines^34^ on 27 September 2024.

A systematic search was conducted on PubMed and Ovid/Embase using the terms “ED shock wave” and “ED shockwave” from the days of inception of these databases until 27 September 2024, according to the 2020 PRISMA (Preferred Reporting Items for Systematic Reviews and Meta-Analyses)^34^ guidelines. The strategy of the assessment of the identified reports is summarized in Fig. 1. For PubMed, these searches retrieved 270 and 199 records, respectively. In Ovid/Embase, both searches led to the proposed subject headings “ED” and “shock wave therapy.” Combining these subject headings in a single search with the Boolean operator AND yielded 110 records. Thus, the total number of records identified from databases was 579, of which 220 were duplicates that were removed before screening. Furthermore, the 16 meta-analyses listed in Table 1 were searched for studies that were not found in PubMed and Ovid/Embase. After removal of abstracts of presentations at scientific conferences, one additional record remained. Automation tools for marking records as ineligible were not used.

The resulting 360 records were screened, and studies on other pathologies than ED (e.g. Peyronie's disease), reviews, commentaries, editorials, letters to the editor, and studies on animal models were excluded (together 268 records). From the 92 reports sought for retrieval (references in Supplementary Data), all but one were downloaded from the E-media library of LMU Munich (Munich, Germany) or obtained through other sources and were assessed for eligibility. Translation from languages other than English was performed using Google Translate. One case series^35^ (*n* = 32 patients; no control group) in Chinese language was not retrieved.

Four of the 91 reports assessed for eligibility were excluded from further analysis. (i) Two publications^36^^,^^37^ were preliminary reports (named clinical trial updates by the authors) of a randomized controlled trial (RCT)^38^ after enrollment of ~25%^36^ or 50%^37^ of the patients into the RCT. (ii) One publication^39^ was a national, multi-institutional, retrospective progress report about the use of four different ESWT devices in the treatment of ED. However, the partially incorrect descriptions of the ESWT devices in this report^39^ raised doubts about the scientific integrity of the entire publication. Specifically, the BTL-6000 SWT device (BTL, Prague, Czech Republic) was described as electropneumatic generator with focused-wave morphology but actually generates radial ESWs (rESWs), whereas the 250 device (Shenzhen Huikang Medical Apparatus Co., Shenzhen, China) was described as electromagnetic (EM) generator with radial-wave morphology but actually generates focused ESWs (fESWs). (iii) In one study,^40^ only ~two-thirds of the patients suffered from ED, without separate reporting of the results of the ED patients.

The remaining 87 reports were included in the systematic review. Forty of them were reports of case series without control groups, 15 were reports of cohort studies with one or more non-randomized control groups, and 32 were reports of RCTs.

Table 2 summarizes the variables that were extracted from the 87 identified reports. For case series, 38 variables were extracted, and for cohort studies and RCTs with one/two/three control groups, 45/50/55 variables were extracted.

**Table 2:** Variables extracted from the 87 reports on ESWT for ED identified in the systematic literature search outlined in Fig. 1

**Name**	**Variable**
**A—General information**	
V1	Name of the first author
V2	Year of publication
V3	The country (or countries) in which the clinical trial was performed were specified (country name(s)/not specified)
V4	The type of investigated ED was specified (vasculogenic/organic/post-radical prostatectomy/diverse/not specified)
V5	The patients were non-responders to PDE5i (yes/no/both/not specified)
V6	The blood testosterone level of all patients enrolled in the trial was normal (yes/no/not specified)
V7	Testosterone replacement therapy was performed in case of testosterone deficiency (yes/no/not applicable)
V8	The IIEF-EF score was determined to enable comparison of treatment outcome with other studies on ESWT for ED, including the first description in the literature (yes/no)
V9	The type of clinical trial was specified (case series/cohort study/RCT)
V10	Control groups were specified (not applicable/1/2/3/etc.)
V11	It was specified whether the data were prospectively or retrospectively collected (prospective/retrospective/not specified)
V12	The time post-baseline or post-treatment [weeks] at which the primary endpoint was determined was specified. (Note: in this systematic review, sometimes data from a different time point were used for analysis in order to keep the times post-treatment as homogeneous as possible.)
V13	Additional follow-up times [weeks or months] were reported (values/not applicable)
**B—ESWT device used**	
V14	The exact name of the used device was specified (name/not specified)
V15	The manufacturer's name, country, and city of the headquarter were specified (data/not specified)
V16	The type of generated ESWs was specified (focused (F)/unfocused (U)/linear (L)/radial (R)/not specified)
V17	The technology of ESW generation was specified EH)/EM/PE/EM-ballistic (EMB)/PB/not specified)
**C—(Main) ESWT group**	
V18	The number of patients in the (main) ESWT group was specified (number/not specified)
V19	The number of patients in the (main) ESWT group lost to follow-up was specified (number/not specified)
V20	The age of the patients [years] in the (main) ESWT group was specified (minimum/median/mean/SD/maximum/interquartile distance/not specified)
V21	The duration of ED before baseline [months] of the patients in the (main) ESWT group was specified (minimum/median/mean/SD/maximum/interquartile distance/not specified)
V22	The number of treatment sessions was specified (number/not specified)
V23	The time sequence of the treatment sessions was specified (weeks and number of sessions per week/not specified)
V24	The number of ESWs per treatment session was specified (number of treatment regions distributed over the penis times number of ESWs per treatment region/not specified)
V25	The type of the reported energy density of the ESWs was specified (ED^+^/ED^total^/not specified whether ED^+^ or ED^total^)
V26	The energy density of the ESWs [mJ/mm^2^] was specified (value/not specified).
V27	Alternatively, there was another description of the energy settings of ESWs (KV/mJ/bar)
V28	The 3D acoustic pressure field of the applied ESWs (i.e. for the device settings used in the clinical trial) was analyzed, including determination of the 3D regional distribution of the peak positive pressure, peak negative pressure, and the resulting energy density (yes/no)
V29	Alternatively, the report referred to a publication in which the 3D acoustic pressure field of the applied ESWs (i.e. for the device settings used in the clinical trial) was analyzed as described in V28 (yes/no)
V30	The frequency of the applied ESWs [Hz] was specified (value/not specified)
V31	Additional treatment(s) next to ESWT were specified (additional treatment(s)/no additional treatment(s))
**D—First control group (if applicable)**	
V32	Data corresponding to V18
V33	Data corresponding to V19
V34	Data corresponding to V20
V35	Data corresponding to V21
V36	The treatment(s) of the patients in the first control group were specified (treatment(s)/not specified)
**E—Second control group (if applicable)**	
V37	Data corresponding to V18
V38	Data corresponding to V19
V39	Data corresponding to V20
V40	Data corresponding to V21
V41	Data corresponding to V36
**F—Third control group (if applicable)**	
V42	Data corresponding to V18
V43	Data corresponding to V19
V44	Data corresponding to V20
V45	Data corresponding to V21
V46	Data corresponding to V36
**G—Statistical analysis**	
V47	An ITT analysis was performed (yes/no)
V48	There were missing data due to patients lost to follow-up (yes/no)
V49	Missing data imputation was performed (yes/no/not applicable as no patient was lost to follow-up)
V50	The method(s) used for missing data imputation were specified (method(s)/not applicable)
V51	An estimand strategy for handling intercurrent events was developed (yes/no)
V52	The reported data are suitable for calculating an average mean ± SD of mean IIEF-EF data reported in different clinical trials at baseline and at follow-up (i.e. mean IIEF-EF values at baseline and at follow-up must be reported) (data)
V53	The data were suitable for a meta-analysis (i.e. mean, SD, and the number of patients in the ESWT group, and the main control group were available at baseline and at follow-up, and the use or non-use of ESWT was the only difference between the groups (sham treatment was considered non-use of ESWT)) (yes/no/not applicable for case series)
**H—Therapeutic outcome**	
V54	ESWT resulted in a statistically significant improvement of ED compared to baseline (yes/no/not applicable)
V55	ESWT resulted in a statistically significant improvement of ED compared to sham or control treatment (not applicable for case series; A, ESWT+, C−, ESWT > C; B, ESWT+, C+, ESWT > C; C, ESWT+, C+, ESWT = C; D, ESWT−, C−, ESWT = C; E, ESWT−, C+, ESWT < C

Statistical analyses (calculation of mean, SD, median, and range of the investigated variables as well as Wilcoxon matched-pairs signed-rank test for effect estimation) were calculated using GraphPad Prism (Version 10.3.1 for Windows; GraphPad Software, Boston, MA, USA). Furthermore, a meta-analysis was performed using the software Comprehensive Meta-Analysis (Version 4; Biostat, Inc., Englewood, NJ, USA). *P*-values smaller than 0.05 were considered statistically significant.

## Results

The 87 eligible studies exhibited considerable heterogeneity in the investigated variables (all extracted values are provided in Supplementary Data).

### Type of erectile dysfunction

In 34 of the 87 (39%) studies, vasculogenic ED was investigated, in 18 (21%) studies organic ED, in 12 (14%) studies diverse kinds of ED, in 10 (11%) studies ED post-radical prostatectomy, and in one (1%) study each ED in the presence of Peyronie's disease, priapism-induced ED, ED on the basis of chronic pelvic pain syndrome, veno-occlusive ED based on hypogonadotropic hypogonadism, post pelvic fractures associated with urethral injury, and multifactorial ED in kidney transplant recipients. In 7 (8%) studies, the type of investigated ED was not specified.

### Collection of data

In 62 of 87 (71%) studies, the data were prospectively collected, and in 15 (17%) studies retrospectively. In 10 (11%) studies, it was not specified whether the data were prospectively or retrospectively collected.

### Response to PDE5i

In 20 of 87 (23%) studies, the patients were non-responders to PDE5i, whereas in 64 (74%) studies, this was not the case. In 3 (3%) studies, both responders and non-responders to PDE5i were included.

### Blood testosterone levels

In 34 of 87 (39%) studies, the blood testosterone level of all enrolled patients was normal. In 3 (3%) studies, this was not the case, and, thus, testosterone replacement therapy was performed in cases of testosterone deficiency. However, in 50 (57%) studies, the blood testosterone level of the enrolled patients was not specified.

### International index of erectile function—erectile domain score

In 37 of the 87 (43%) studies, the International Index of Erectile Function—Erectile Domain (IIEF-EF) score^41^ was determined to enable comparison of treatment outcome with other studies on ESWT for ED, including the first description in the literature.^42^ In 50 (57%) studies, this was not the case.

### Number of patients

The number of patients in the (main) ESWT group varied between 5 and 710 (mean, 55; SD, 87; median, 35), and the number of patients in the first (main) control group varied between 10 and 484 (mean, 45; SD, 68; median, 34). In six studies, a second control group was investigated in which the number of patients varied between 24 and 178 (mean, 72; SD, 68; median, 34.5). Furthermore, in one study, a third control group with 25 patients was investigated.

### Age of the patients

The average age of the patients could not be calculated, as several studies reported only median data. The average of the 64 of 87 (74%) reported mean ages of patients in the (main) ESWT group was 53.3 years ±8.6 years (mean ± SD), and the average of the 22 (25%) reported median ages of patients in the (main) ESWT group was 58.8 years ±3.8 years. Furthermore, in 37 (43%) studies, the range of the age of the patients in the (main) ESWT group was specified; the lower limit varied between 19 years and 55 years (mean, 36 years; SD, 11 years; median, 33 years), and the upper limit varied between 36 years and 84 years (mean, 71 years; SD, 9 years; median, 72 years). In one study, the age of the patients was not specified.

### Duration of erectile dysfunction before baseline

The average duration of ED before baseline of the patients in the (main) ESWT group could neither be calculated, as several studies reported only median data. However, the following information could be extracted from the provided data: (i) in 28 of 77 (36%) studies, addressing ED other than post-prostatectomy the mean duration of ED before baseline was specified, with values varying between 5.5 months and 118 months (mean, 43.8 months; SD, 27.7 months; median, 34.6 months); (ii) in 16 of 77 (21%) studies, the median duration of ED before baseline was specified; these values varied between 12 months and 68 months (mean, 47.1 months; SD, 15.8 months; median, 46 months); (iii) in 19 of 77 (25%) studies, a lower limit of the duration of ED before baseline was reported (3× >3 months, 14× > 6 months, and 2× >12 months), and in 1 of 77 (1%) studies an upper limit (<6 months); (iv) in 23 of 77 (30%) studies, the range of the duration of ED before baseline was specified; the lower limit varied between 3 months and 36 months (mean, 10.8 months; SD, 7.2 months; median, 9 months), and the upper limit varied between 12 months and 240 months (mean, 116 months; SD, 82 months; median, 84 months); and (v) in 13 of 77 (17%) studies, the duration of ED before baseline was not specified.

### Extracorporeal shock wave therapy devices used

**Figure 2:**
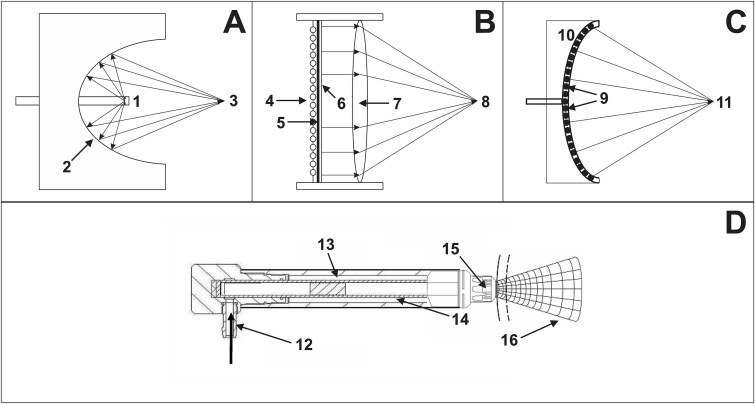
Schematic of focused (A–C) and radial (D) extracorporeal shock wave generators (adapted from,^11^ CC BY-NC license). (A) EH principle (fESWs): A high-voltage spark discharge between two electrodes (1) in water creates a gas bubble filled with vapor and plasma. Its rapid expansion produces a sonic pulse, and its implosion creates a reverse shock wave. Reflectors (2) focus this wave to a second focal point (3), generating high-pressure acoustic energy. (B) Electromagnetic principle (fESWs): A strong magnetic field from an electric current in a coil (4) induces a current in a metal membrane (5), which rapidly propels an adjacent conductive membrane (6) in fluid. This generates a shock wave, focused via an acoustic lens (7) to a focal point (8). (C) PE principle (fESWs): Multiple piezocrystals (9), typically 1000–2000, are mounted in a bowl-shaped device (10). A rapid electrical discharge causes them to deform, emitting acoustic pulses that steepen into shock waves. The bowl shape focuses the wave at a focal point (11). (D) Ballistic principle (rESWT): Compressed air (12) or a magnetic field propels a projectile (13) through a guiding tube (14) to strike a metal applicator (15) on the skin, producing mechanical stress waves transmitted into tissue (16). Linear ESWs can be generated by serially arranging multiple units like (C).

The electrohydraulic (EH), EM, piezoelectric (PE), and ballistic principles for generating ESWs are schematically shown in Fig. 2 (taken from^11^ which was published under the Creative Commons CC-BY-NC license).

In 22 of the 87 (25%) studies, the focused part of the EM device, Duolith SD1 (fESWs; Storz Medical, Tägerwillen, Switzerland) was used; in 18 (21%) studies, the EH device, Omnispec ED1000 (fESWs; Medispec, Yehud, Israel) was used; in 10 (12%) studies, the EM device, Renova (linear ESWs (lESWs); Direx, Petah Tikva, Israel) was used; in 9 (10%) studies, the PE device, Piezowave 2 with FBL10x5G2 handpiece (lESWs; Richard Wolf, Knittlingen, Germany) was used; in 7 (8%) studies, the EM device, Aries 2 (fESWs; Dornier MedTech, Weßling, Germany) was used, and in 2 (2%) studies, the EH device, UroGold 100 (fESWs; MTS, Konstanz, Germany) was used. Furthermore, in one (1%) study, each the following devices were used: BTL-6000 SWT (pneumatic-ballistic (PB); rESWs; BTL); enPulse Pro (EM-ballistic (EM-B); rESWs, Zimmer, Neu-Ulm, Germany); ESWO-I 80 mm (EM; fESWs; Shenzhen Hyde Medical Equipment Co, Shenzhen, China); GentlePro (EM-B; rESWs; Zimmer); HB-ESWT−01 (not specified; fESWs; Zhanjiang Haibin Medical Equipment Co, Guangdong, China); Intellect Focus Shockwave Therapy SKU (EM; fESWs; Chattanooga, Chattanooga, TN, USA); LGT-2510B (P-B; rESWs; Guangzhou Longest Medical Technology Co, Guangzhou, China); Masterpuls MP50 (P-B; rESWs, Storz Medical); MoreNova (EM; lESWs; Direx); MT 2000H (EM; fESWs; Urontech, Hwaseong, Korea); OrthoGold 100 (EH; fESWs; MTS, Konstanz, Germany); Piezowave 2 with FB10G6 handpiece (PE; fESWs; Richard Wolf); Swiss DolorClast with EvoBlue Handpiece (P-B; rESWs; Electro Medical Systems); and WellWave (PE; fESWs; Richard Wolf). In 5 (6%) studies, the used device was not specified.

It should be mentioned that in one study^43^ in which the Duolith SD1 (Storz Medical) was used, a linearly formed transducer head was described without providing further details. It is unknown whether this linearly formed transducer head generated lESWs similar to the Renova (Direx), MoreNova (Direx), and the Piezowave 2 with FBL10×5G2 handpiece (Richard Wolf), or whether this was a regular fESW transducer head with a standoff with an elongated recess/trough. In the other 21 studies in which the Duolith SD1 (Storz Medical) was used, a linearly formed transducer was not mentioned. As in the same study,^43^ the patients were also treated with a curved transducer head (which was probably the regular handpiece of the Duolith SD1 (Storz Medical) that generates fESWs), this study was considered focused ESWT in this systematic review.

### Types of extracorporeal shock wave applied

In 56 of the 87 (65%) studies, fESWs were applied, in 20 (23%) studies, lESWs were applied, in 6 (7%) studies, rESWs were applied, and in 1 (1%) study, unfocused ESWs were applied. In 4 (5%) studies, the type of ESWs was not specified or could not be determined by the description of the used device.

### Treatment protocols

The treatment protocols were analyzed both cumulatively and separately for studies in which fESWs, lESWs, and rESWs were applied (details of the separate analysis are summarized in Table 3). The most commonly used treatment protocols were Week 1 (W1) to W3 and W7–W9 with one treatment session per week (Protocol A; 16 of 87 (18%) studies), W1–W4 with one treatment session per week (Protocol B; 12 (14%) studies), and W1–W6 with one treatment session per week (Protocol C; also 12 (14%) studies). Various other treatment protocols were used. Of note, Protocols A/B/C were the most commonly used treatment protocols among those studies in which fESWs/lESWs/rESWs were applied.

**Table 3:** Details of the treatment protocols of ESWT for ED used in the 87 clinical trials identified in this systematic review, stratified by the type of the applied extracorporeal shock waves

	**fESWs**	**lESWs**	**rESWs**
TS	9.0 ± 3.3 (9; 4–18) [56]^a^	5.6 ± 2.2 (4.5; 4–10) [20]^a^	6.7 ± 1.0 (6; 6–8) [6]^a^
ESWs/TS	2773 ± 1556 (3000; 1500–10 000) [55]^a^	3746 ± 1758 (3800; 600–6600) [20]^a^	5000 ± 2828 (4500; 2000–10 000) [6]^a^
∑ESWs	24 558 ± 16 690 (18 000; 8000–90 000) [55]^a^	18 980 ± 9633 (18 600; 3600–39 600) [20]^a^	33 000 ± 17 697 (27 000; 12 000—60 000) [6]^a^
EnD [mJ/mm^2^]	0.14 ± 0.07 (0.09; 0.05–0.25) [47]^a^	0.11 ± 0.03 (0.09; 0.09–0.16) [17]^a^	0.09 ± 0.0 (0.09; 0.09–0.09) [2]^a^
∑EnD [J/mm^2^]	3.25 ± 2.46 (1.8; 0.81–9) [47]^a^	2.19 ± 1.44 (1.8; 0.54–6.34) [17]^a^	1.89 ± 1.15 (1.9; 1.1–2.7) [2]^a^
F [Hz]	3.8 ± 1.8 (3.7; 1.7–8) [44]^a^	5.3 ± 1.8 (5; 2–8) [9]^a^	13.7 ± 2.9 (12; 12–17) [3]^a^
∑TT [min]	119 ± 66 (116; 33–400) [44]^a^	58 ± 45 (48; 20–167) [9]^a^	41 ± 23 (33; 24–67) [3]^a^
P	W1–W3 and W7–W9 (1× per week) [13]^a^	W1–W4 (1× per week) [9]^a^	W1–W6 (1× per week) [3]^a^

Data are provided as mean ± SD (median; range).

^a^ The numbers in brackets indicate the numbers of reports that provided the corresponding raw data.

EnD, energy density; ESWs/TS, number of extracorporeal shock waves per therapy session; F, frequency at which the ESWs were applied; fESWs, focused extracorporeal shock waves; lESWs, linear extracorporeal shock waves; P, the most commonly used treatment protocol; rESWs, radial extracorporeal shock waves; TS, number of therapy sessions; total number of applied extracorporeal shock waves (TS × ESWs/TS); W, week; ∑ESWs, ∑EnD, cumulated energy density applied (∑ESWs × EnD); ∑TT, total treatment time (∑ESWs/F).

### Details of the treatment protocols

The number of treatment sessions varied between 4 and 14 (mean, 8.3; SD, 3.3; median, 6; data from 87 studies).

The number of ESWs applied per treatment session varied between 600 and 10 000 (mean, 3022; SD, 1743; median, 3000; data from 86 studies, as not in all studies all relevant information was provided).

The total number of applied ESWs varied between 3600 and 90 000 (mean, 23 120; SD, 14 684; median, 18 000; data from 86 studies).

The energy density of the applied ESWs varied between 0.05 millijoule (mJ)/mm^2^ and 0.25 mJ/mm^2^ (mean, 0.13 mJ/mm^2^; SD, 0.06 mJ/mm^2^; median, 0.09 mJ/mm^2^; and data from 71 studies). Obviously, incorrect information about the energy density of the applied ESWs (0.009 mJ/mm^2^ as well as 20 mJ/mm^2^, 15 mJ/mm^2^, and 12 mJ/mm^2^)^44–46^ was not considered in these calculations. The same applied to other descriptions of the energy settings of ESWs (including kilovolt (KV), mJ, and bar)^47–51^ from which, without further information, no direct conclusions can be drawn about the energy density of the applied ESWs.

The cumulated energy density applied over all treatment sessions varied between 0.54 J/mm^2^ and 9 J/mm^2^ (mean, 2.83 J/mm^2^; SD, 2.15 J/mm^2^; median, 1.8 J/mm^2^; data from 71 studies).

The frequency of the applied ESWs varied between 1.66 Hz and 17 Hz (mean, 4.3 Hz; SD, 2.7 Hz; median, 4 Hz; data from 58 studies).

From these values, the total treatment time was calculated (total number of applied ESWs divided by the frequency at which the ESWs were applied) that varied between 20 min and 500 min (mean, 111 min; SD, 84 min; median, 100 min; data from 60 studies).

Of note, none of the 87 studies specified whether the reported energy density of the ESWs represented the positive or total energy density. Furthermore, no study analyzed the 3D acoustic pressure field of the applied ESWs (i.e. for the device settings used in the study), including the determination of the 3D regional distribution of the peak positive pressure, peak negative pressure, and the resulting energy density. Furthermore, none of the studies referenced any publication that provided an analysis of the 3D acoustic pressure field for the ESW device settings used.

### Combination treatments

In 27 of the 87 (31%) studies, ESWT was combined with other treatments. These other treatments were PDE5i oral (daily or on demand; 19 studies) and PDE5i oral + L arginine supplement (2 studies), as well as application of ESWs to the pelvic floor, pelvic floor training, transcranial magnetic stimulation, use of a vacuum erectile device, injection of platelet-rich plasma, and subcutaneous injection of recombinant chorionic gonadotropin plus oral intake of *Epimedium Brevicornum* (1 study each). In 60 (69%) studies, ESWT was not combined with other treatments.

### Intent-to-treat analysis

In 51 of the 87 (59%) studies, an intent-to-treat (ITT) analysis^52–54^ was performed, whereas in 36 (41%) studies, this was not the case. Furthermore, in 38 (44%) studies, at least one patient was lost to follow-up in any of the investigated groups, with group-specific lost to follow-up rates varying between 2.3% and 100% (mean, 19.7%; SD, 16.8%; median, 16.7%). However, in only 2 of the 38 (5%) studies with missing data, missing data imputation^55^^,^^56^ was performed (using the baseline observation carried forward (BOCF) method).

### Estimand strategy for handling intercurrent events

In only 1 of the 87 (1%) studies, an estimand strategy for handling intercurrent events^57–59^ was developed.

### Treatment outcome

Among the 77 studies that did not address ED post-prostatectomy, 71 (92%) studies reported a statistically significant improvement in ED from baseline to follow-up. Furthermore, 33 of these 77 (43%) studies reported mean IIEF-EF values at baseline and at follow-up. The mean IIEF-EF values at baseline varied between 7 and 21.2 (mean, 13.7; SD, 3.4; median, 14), and the mean IIEF-EF values at follow-up varied between 12.3 and 25.8 (mean, 19.5; SD, 3.6; median, 20). Statistical analysis using the Wilcoxon matched-pairs signed-rank test demonstrated a significant (*P* < 0.001) difference in the mean IIEF-EF values at baseline and at follow-up. The mean follow-up time in these 33 studies varied between 7 and 30 weeks (mean, 18.1 weeks; SD, 7.1 weeks; median, 24 weeks).

**Table 4:** Details of the meta-analysis of 19 cohort studies and RCTs on ESWT for ED performed in this systematic review

**Ref**	**Study**	**T**	**SD-M**	**SE**	**V**	**LL**	**UL**	***Z***	***P***
^51^	Trishch et al. (2024)	CS	2.384	0.317	0.101	1.762	3.005	7.515	<0.001
^60^	Karakose et al. (2021)	CS	2.813	0.347	0.121	2.132	3.493	8.099	<0.001
^61^	Verze et al. (2020)	CS	1.137	0.173	0.03	0.799	1.476	6.591	<0.001
	Pooled (all CSs)		2.082	0.564	0.318	0.976	3.187	3.692	<0.001
^62^	Kalyvianakis et al. (2024)	RCT	1.547	0.334	0.112	0.892	2.203	4.63	<0.001
^63^	Kennady et al. (2023)	RCT	0.884	0.359	0.129	0.18	1.588	2.46	0.014
^64^	Kalyvianakis et al. (2022)	RCT	1.737	0.281	0.079	1.188	2.287	6.193	<0.001
^65^	Motil et al. (2022)	RCT	0.315	0.318	0.101	−0.308	0.939	0.99	0.322
^66^	Ong (2022)	RCT	0.927	0.295	0.087	0.349	1.506	3.141	0.002
^47^	Sand.-Salinas et al. (2022)	RCT	0.25	0.224	0.05	−0.19	0.69	1.114	0.265
^67^	Ladegaard et al. (2021)	RCT	0.749	0.336	0.113	0.091	1.408	2.23	0.026
^68^	Shendy et al. (2021)	RCT	1.463	0.347	0.121	0.782	2.144	4.212	<0.001
^46^	Kim et al. (2020)	RCT	2.433	0.271	0.073	1.903	2.964	8.987	<0.001
^69^	Sramkova et al. (2019)	RCT	6.565	0.653	0.426	5.286	7.844	10.06	<0.001
^70^	Yamaçake et al. (2019)	RCT	0.877	0.468	0.219	−0.041	1.794	1.872	0.061
^71^	Fojecki et al. (2017)	RCT	0.461	0.181	0.033	0.107	0.815	2.554	0.011
^72^	Kalyvianakis et al. (2017)	RCT	1.224	0.335	0.112	0.568	1.88	3.655	<0.001
^73^	Srini et al. (2015)	RCT	3.339	0.277	0.077	2.796	3.882	12.047	<0.001
^74^	Yee et al. (2014)	RCT	0.366	0.241	0.058	−0.107	0.838	1.516	0.129
^75^	Vardi et al. (2012)	RCT	0.749	0.271	0.074	0.218	1.281	2.762	0.006
	Pooled (all RCTs)		1.424	0.278	0.007	0.879	1.969	5.120	<0.001
	Pooled (all studies)		1.526	0.244	0.059	1.048	2.004	6.259	<0.001

CS, cohort study; LL, lower limit; Ref, reference; RCT, randomized controlled trial; SD-M, standard difference in means; SE, standard error; T, type of study; UL, upper limit; V, variance; Z, *Z*-value; P, *P*-value.

In 20 of the 47 (43%) cohort studies and RCTs, ESWT was statistically significantly superior to sham/control treatment. Furthermore, in 5 (11%) of these studies, ESWT and sham/control treatment significantly improved ED, but ESWT was not significantly superior to sham/control treatment. In only 2 (4%) of these studies, neither ESWT nor sham/control treatment was effective, and in none of these studies, sham/control treatment was significantly superior to ESWT. For the remaining 20 cohort studies and RCTs, the corresponding analysis could not be performed based on the published data.

### Meta-analysis

**Figure 3:**
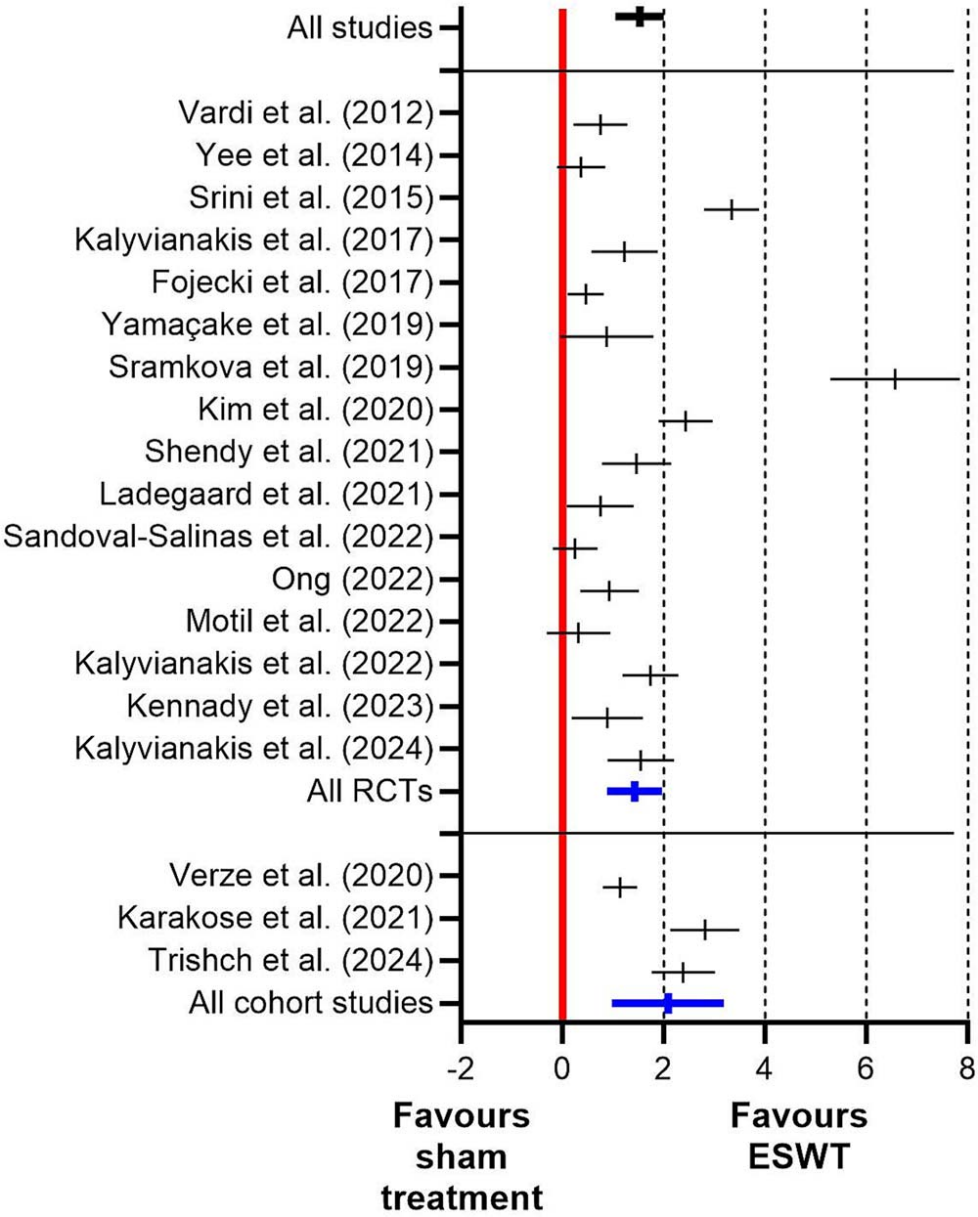
Results of the meta-analysis of 19 cohort studies and RCTs on ESWT for ED performed in this systematic review.

Nineteen of the 47 (40%) cohort studies and RCTs were suitable for a meta-analysis (i.e. mean and SD of the primary endpoint (e.g. the IIEF-EF score) as well as the number of patients in the (main) ESWT group and the main control group were reported at baseline and at follow-up, and the use or non-use of ESWT was the only difference between the groups (sham treatment was considered non-use of ESWT)). The details of the meta-analysis are summarized in Table 4 and Fig. 3. For the pooled analysis, the standard difference in means was 1.53 (variance; 0.06; lower limit, 1.05; upper limit, 2.00; *Z*-value, 6.26; *P* < 0.001).

### Safety

In none of the 87 studies, severe adverse events were reported.

## Discussion

The results of this systematic review can be summarized and interpreted as follows: (i) there is clear evidence that ESWT for ED is effective and safe, making it a valuable treatment modality in ED management; (ii) the AUA’s assessment that the true effect of ESWT for ED may differ substantially from current estimates is valid; (iii) without a fundamental shift in the planning, implementation, and analysis of clinical studies on ESWT for ED, the AUA’s evaluation is unlikely to change; and (iv) future studies on ESWT for ED should incorporate rigorous characterization and reporting of ESWs, appropriate handling of missing data and intercurrent events, and comprehensive classification of ESWT within the broader landscape of available ED treatment options.

In the following paragraphs, each of these aspects is examined in detail.

### Adequate characterization and reporting of extracorporeal shock waves

**Figure 4:**
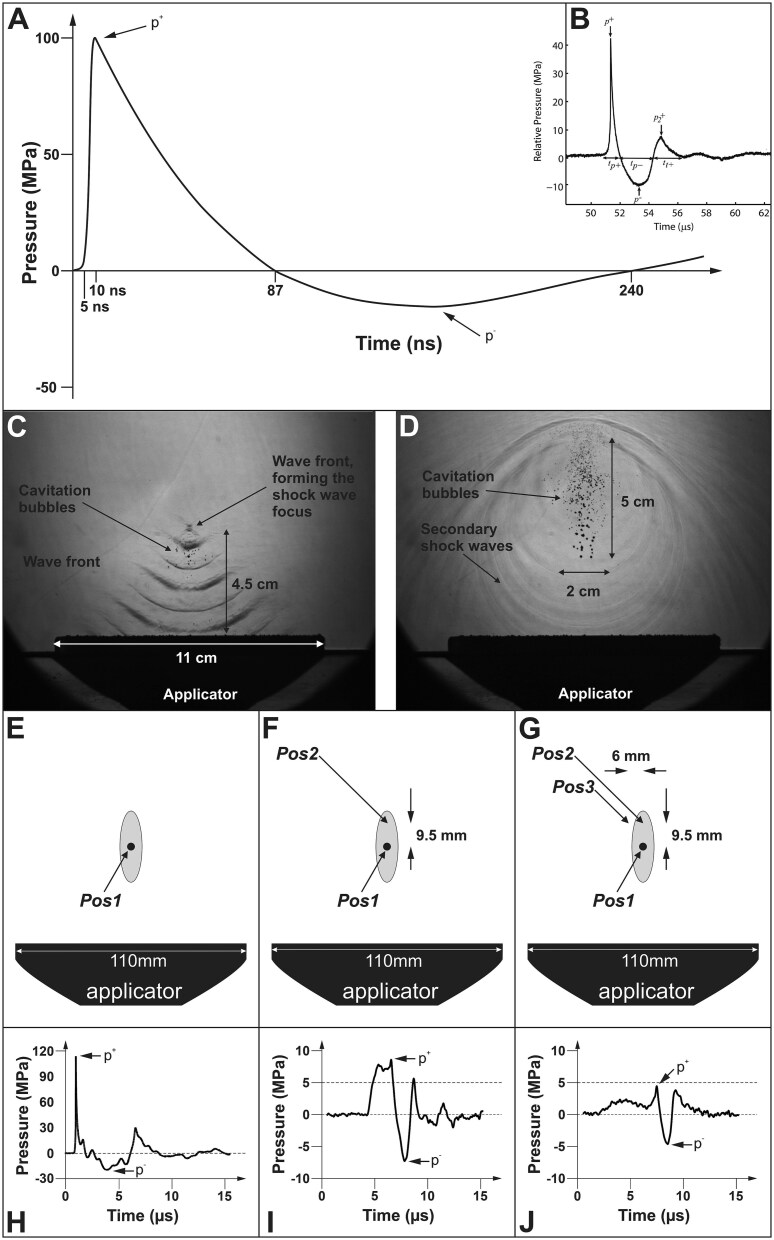
Representative pressure vs time plots and shadow imaging of extracorporeal shock waves for illustration purposes. Panel B was taken from^77^ with permission^78^; Panels C and D were taken from^79^, which was published under the Creative Commons CC BY 2.0 license; and Panels E–J were taken from^80^, which was published under the Creative Commons CC BY license. Pos, position. Details are in the main text.

The shock wave energy profiles employed in ESWT for ED are crucial for understanding the treatment’s effectiveness. In this systematic review, we highlight significant discrepancies between the theoretical profiles of ESWs described in the literature and those used in clinical studies. Figure 4A, redrawn from a recent review on shock wave physics,^76^ illustrates an idealized pressure–time curve for ESWs at a single point along their trajectory. However, it is important to emphasize that none of the patients treated for ED in the 87 studies identified in our systematic review were exposed to ESWs with the energy signature depicted in Fig. 4A.

To demonstrate the difference, Fig. 4B presents an averaged pressure–time curve from 25 ESWs generated by the Duolith SD1 (Storz Medical) at its maximum output setting. This device, which was used in 22 of the studies included in our systematic review, shows substantial differences compared to the idealized curve in Fig. 4A: (i) a much lower peak positive pressure (~43 MPa vs. 100 MPa), (ii) a steeper decrease in positive pressure, and (iii) a significantly longer negative pressure phase (~2.3 μs vs. 150 ns). Note: the ESWs generated by the Duolith SD1 (Storz Medical) do not meet the strict definition of a true shock wave.^77^ As shown in a prior study, shock formation did not occur at any setting of the Duolith SD1 (Storz Medical), and simulations suggested that doubling the initial pressure output would be necessary for generating a true shock wave in water.^77^ Consequently, ESWs generated by the Duolith SD1 (Storz Medical) are better characterized as focused pressure waves rather than true shock waves. This is important because (at least in the context of using the Duolith SD1 (Storz Medical)) there is no scientific justification for distinguishing between focused shock waves and radial pressure waves in ESWT for ED (as done in, e.g.^47^^,^^76^), despite the fact that fESWs and rESWs have different energy signatures.^11^^,^^79^^,^^81^^,^^82^

**Figure 5:**
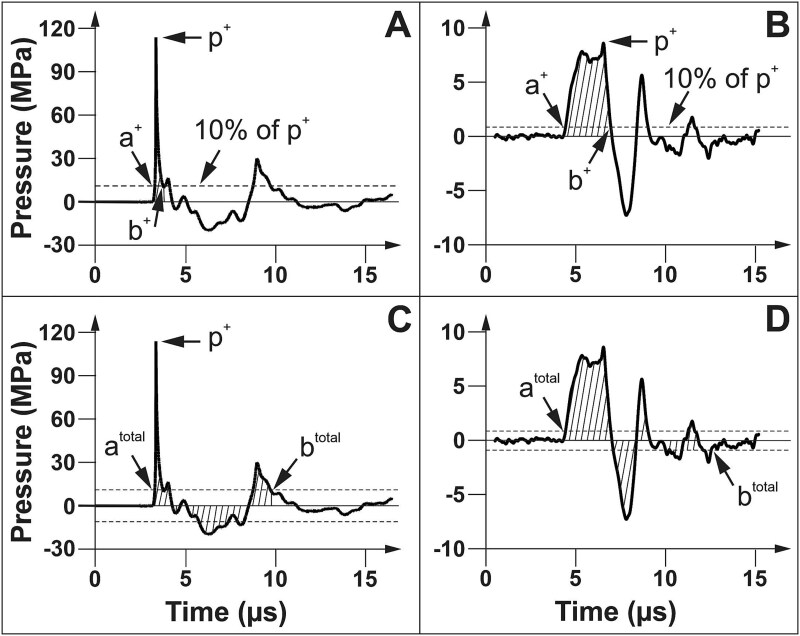
Calculation of the energy density of an extracorporeal shock wave. Pressure vs. time plots in (A and C) are the same as in Fig. 4H, and those in (B and D) are the same as in Fig. 4I. The energy density of an ESW is given by the equation $\mathrm{Energy}\ \mathrm{density}=\frac{1}{Z}{\int}_a^bp{(t)}^2 dt$, where *Z* represents the impedance of sound in water, and *p*(*t*) is the pressure as a function of time, with (a) and (b) being the temporal integration limits. According to IEC-61846,^84^ there are two types of integration limits: positive temporal integration limits and total temporal integration limits. The positive temporal limits are defined as the times between which the positive pressure first exceeds 10% of *p* + (at a+, as shown in A and B) and the first time it falls below 10% of *p* + (at b+, as shown in A and B). In contrast, the total temporal integration limits are the times during which the absolute value of the pressure pulse waveform exceeds 10% of *p* + ​ (at a^total^, as shown in C and D) and the last time it drops below 10% of p + (at b^total^, as shown in C and D). To clarify, the hatched areas under the pressure vs. time plots in A and B represent positive energy density, while the hatched areas in C and D represent total energy density.

These findings raise an important question: are all ESWT treatments for ED characterized as focused ESWT actually performed with focused pressure waves? The current literature, including reviews of ESWT for ED,^76^^,^^83^ does not provide sufficient data to resolve this issue. In any case, even if a device capable of generating true shock waves is used, it is unlikely that the entire target tissue would receive uniform treatment. This is due to the fact that the 3D acoustic pressure field generated by ESWT devices is not homogeneous, leading to variations in pressure and energy density across different tissue regions (the calculation of the energy density of an ESW at one point of its trajectory according to IEC-61846^84^ is illustrated in Fig. 5).

Figures 4C and D illustrate the acoustic pressure field of an ESWT device (PiezoClast; Electro Medical Systems) that has not been used in published clinical trials on ESWT for ED. Using shadow imaging,^85^ the elliptical field of cavitation bubbles^86^^,^^87^ in Fig. 4D already indicates the absence of a homogeneous acoustic pressure field above the applicator of the ESWT device used. These observations highlight the inherent complexity of the 3D acoustic pressure field generated by ESWT devices, which affects how energy is distributed across tissue.

The pressure–time plots shown in Fig 4E–J further demonstrate the heterogeneity of the 3D acoustic pressure field of ESWs (demonstrated here for the PiezoClast (Electro Medical Systems)). As pressure measurements at different positions within the acoustic pressure field showed, the peak positive and negative pressures varied significantly across the 3D acoustic pressure field. Notably, the pressure profile at the focus point (Position 1 in Fig. 4E) exhibited much higher peak pressures (p^+^ ~ 110 MPa, p^−^ ~ −20 MPa; Fig. 4H) compared to other locations, such as Positions 2 and 3 in Fig 4F and G, where pressures were substantially lower (shown in Fig 4I and J). These measurements underscore the varying energy densities to which tissues are exposed within the treatment zone.

The non-homogeneous nature of the 3D acoustic pressure field implies that energy densities vary significantly within the 3D space affected by ESWs. The region around the focus point, where the local peak positive pressure (p^+^) is at least 50% of the value at the focus point, is defined as the −6 dB focus, and a 5 MPa focus is defined as the region where the local p^+^ exceeds 5 MPa, irrespective of the pressure at the focus point.^84^^,^^88^ Understanding these variations is essential for optimizing treatment protocols for ED, as exposure to different pressure conditions could lead to varying biological effects.

This variability in the 3D acoustic pressure field and, thus, the spatial distribution of the energy density of ESWs, raises important questions about the potential effects of ESWs on target tissues, particularly cells within the treated region. The shear stress induced by positive pressure and the cavitation caused by negative pressure may have distinct effects on cellular and tissue responses. Although only a few studies have explored the impact of cavitation on cells,^89–91^ these experiments suggest that cavitation plays a significant role in the biological effects of ESWT, with varying effects depending on the medium (e.g. water or high-viscosity solutions) and the type of tissue exposed.^92^

It is essential to recognize that the size of cells relative to the spatial characteristics of ESWs suggests that cells may not distinguish between focused and radial shock waves. However, variations in pressure, energy density, and the duration of exposure to positive and negative pressures could lead to significantly different cellular responses. Thus, cells within different regions of the 3D acoustic pressure field of ESWs may experience distinct mechanical forces, influencing their biological behavior and response to treatment.

Furthermore, the specification of energy density in clinical studies is often ambiguous, with several publications reporting the input energy in KV,^48^ mJ,^49^^,^^51^ or bar values,^47^^,^^50^ rather than the actual energy density of the ESWs delivered. These values describe the energy used to generate ESWs, not the energy density experienced by tissues, making it difficult to draw meaningful conclusions from reported KV, mJ, and bar data. Moreover, discrepancies between reported energy densities and actual measured values highlight the challenges in reproducing and comparing results across studies.^93^

In conclusion, the spatial and temporal characteristics of the 3D acoustic pressure fields generated by ESWT devices for ED are highly complex and not fully understood. Devices that operate at the same energy density at the focus point may expose tissues to very different acoustic conditions. The need for precise characterization of these 3D acoustic pressure fields is critical for optimizing ESWT protocols and improving the clinical outcomes of ED treatment. Future studies should focus on better defining the 3D acoustic pressure fields, energy densities, and their effects on tissue, ultimately leading to more effective and reproducible treatment strategies for ED. Additionally, collaboration between researchers and device manufacturers will be essential to advancing the field and enhancing the understanding of the biological mechanisms of ESWT.

### Appropriate handling of missing data and intercurrent events

Missing data can have substantial impact on the results—and, thus, the interpretation of the results—of clinical trials. This is particularly the case when data are not missing at random. For example, if patients drop out of a clinical trial during the follow-up period because they are dissatisfied with the treatment outcome, only data from satisfied patients will be collected, which may bias the final results. While missing data is a common challenge in clinical trials, the reasons for missing data can vary significantly across groups, requiring tailored imputation strategies.

More broadly, clinical trials investigating ED treatments should adhere to an ITT analysis,^52–54^ which includes all patients regardless of whether they completed the study. ITT analysis is crucial for preserving the randomized nature of the study and ensuring that the results reflect the real-world effectiveness of the intervention.^94–98^ Moreover, estimand strategies for handling intercurrent events—i.e. events that occur after treatment initiation and either affect the observation of a variable or its interpretation—should be explicitly defined in the study protocol.^99^ Although a detailed discussion of estimand strategies is beyond the scope of this review, it is well-established in the literature that handling intercurrent events through a clearly outlined approach is crucial for drawing valid conclusions from clinical trials.^99^

There are various methods for managing missing data, which can be categorized as missing completely at random, missing at random, or missing not at random.^55^ Each type of missingness requires different imputation strategies. Common approaches include BOCF, last observation carried forward, expectation maximization algorithms, and multiple imputation methods such as the treatment-based Monte Carlo Markov Chain.^56^^,^^55^ Furthermore, the concept of estimands in clinical trials involves four essential components: population, variables, intercurrent events, and population-level summary.^57–59^ There are several strategies to handle intercurrent events, including: (i) treatment policy strategy—data are analyzed regardless of the occurrence of intercurrent events; (ii) composite strategy—the occurrence of the intercurrent event is incorporated into the endpoint; (iii) hypothetical strategy—treatment effects are estimated, assuming that the intercurrent event did not occur; (iv) principal stratum strategy—treatment effects are estimated within the subset of patients who did not experience the intercurrent event; and (v) while-on-treatment strategy—data are analyzed up to the point of the intercurrent event.^57^^,^^58^^,^^99^

Unfortunately, our systematic review of 87 studies on ESWT for ED revealed significant gaps in addressing missing data and intercurrent events. None of the studies developed detailed strategies for handling intercurrent events, and the term “estimand” was absent from all studies. Furthermore, in 44% of the studies, patients were lost to follow-up, but only two studies attempted missing data imputation.^100^^,^^101^ These shortcomings highlight a major obstacle in determining the true effectiveness of ESWT for ED.

For example, in one study of 710 ED patients treated with ESWT, 42% were lost to follow-up, and no attempt was made to impute missing data.^102^ In another study, 15% (ESWT group) and 34% (control group) of patients were lost to follow-up, yet missing data imputation was not performed.^103^ In a third study, imputation was performed using the BOCF method, but this led to misleading results, as patients who were not treatment responders were simply carried forward without any actual follow-up data.^100^

These examples emphasize the need for more rigorous ITT analyses and the development of estimand strategies in clinical studies on ESWT for ED. The lack of such strategies may directly contribute to the inconclusive or misleading findings reported in many of the studies reviewed. This issue could explain why organizations such as the AUA classify ESWT as investigational (Evidence Level C), despite its potential clinical benefits.

Moreover, missing data imputation and handling intercurrent events are not insurmountable challenges. Statistical software, such as R,^104^^,^^105^ offers various imputation methods that can be easily implemented, even in studies with complex missing data patterns. A more detailed and systematic approach to these analyses would improve the quality and interpretability of clinical trials for ED treatments.

Finally, long-term studies comparing the efficacy of ESWT to other treatment modalities in the management of ED would benefit from using survival analysis techniques, such as the Kaplan–Meier estimator,^106^ to evaluate the fraction of patients achieving specific IIEF-EF scores over time. This approach has been successfully used in oncology and could be adapted for ED studies to provide a clearer picture of the long-term effects of ESWT for ED.

### Classification of extracorporeal shock wave therapy in the overall context of the available treatment options for erectile dysfunction

The integration of ESWT with other treatment modalities for ED remains in its early stages. A recent comprehensive review^3^ outlined several treatment options for ED, with the number of studies in our systematic review combining these treatment options with ESWT as follows: phosphodiesterase type 5 inhibitors (PDE5i) (21 studies; 24%), physiotherapeutic exercises (1 study; 1%), L-arginine supplementation (2 studies; 2%), and vacuum pump rehabilitation (1 study; 1%). Notably, no studies were identified in our systematic review that combined ESWT with intracavernosal self-injection therapy, medicated urethral systems for erection, lifestyle modifications, L-citrulline supplementation, ginseng, Vitamin D, curcumin, or psychotherapy/counseling.

Developing a comprehensive strategy for ESWT combination therapies in ED management is beyond the scope of this review. Such an endeavor would necessitate a detailed exploration of the relationship between specific ED subtypes (e.g. vasculogenic, post-prostatectomy) and the molecular and cellular mechanisms by which ESWs and other treatment modalities interact with connective, muscle, and nerve tissues. However, it is important to note that the mere identification of a mechanism of action in a study does not necessarily imply clinical relevance to the observed therapeutic effects.^13^

Future clinical studies on ESWT for ED should consistently consider patients' blood testosterone levels, as omitting this factor may lead to outdated and incomplete evaluations. Additionally, pelvic floor muscle training should be routinely incorporated with ESWT, given its potential to enhance therapeutic outcomes by addressing key anatomical and functional components of erectile function. Considering ESWT’s well-established efficacy in treating functional and ultrastructural muscle injuries,^107^ targeting pelvic floor muscles with ESWs may further optimize treatment outcomes.

In conclusion, the potential for combining ESWT with other ED treatment modalities is vast, with no current evidence suggesting mechanistic conflicts, as seen in other conditions (e.g. ESWT with muscle relaxants for chronic low back pain,^108^ or ESWT with diclofenac for structural muscle injuries^109^). However, the consistent implementation of the other two aspects of ESWT for ED discussed here (adequate characterization of ESWs, appropriate handling of missing data, and intercurrent events) seems to us at present to be more important than considering possible combination therapies.

### Limitations

The primary limitation of this systematic review is the lack of discussion on the impact of substantial heterogeneity among the investigated variables—such as ED type, response to PDE5i, baseline IIEF-EF scores, patient age, duration of ED prior to treatment, type of applied ESWs, and treatment protocol details—on treatment outcomes across the 87 assessed studies. It is well recognized that (i) younger patients may respond differently to ESWT for ED than older patients; (ii) post-prostatectomy ED and vasculogenic ED have distinct pathophysiologies that may necessitate different treatment approaches; and (iii) patients with a 20-year history of ED may experience different treatment outcomes compared to those with only a few months of ED. However, reported differences in treatment outcomes may be significantly influenced by variations in the 3D acoustic pressure fields of the applied ESWs—even when energy density specifications appear identical—as well as by inadequate handling of missing data and intercurrent events. Before analyzing the impact of ED subtypes, patient subgroups, and treatment protocols on ESWT outcomes, it is essential to first address these methodological inconsistencies, particularly when considering combination therapies.

## Conclusion

Current assessments of ESWT for ED as investigational (e.g. by the AUA; Evidence Level: Grade C) may not necessarily stem from a lack of clinical studies, insufficient related basic science, or an inadequate number of systematic reviews and meta-analyses. Instead, the primary deficiencies lie in the scientific quality of the clinical studies published to date, as detailed in this systematic review. We hypothesize that meaningful advancements in this field will only occur if future clinical studies rigorously address key aspects, including the adequate characterization of ESWs, appropriate handling of missing data and intercurrent events, and a comprehensive classification of ESWT within the broader landscape of available ED treatments.

Conversely, the consistent implementation of these principles has the potential to establish ESWT as the first truly regenerative therapy for ED. The pursuit of this goal is justified by the potential benefits to patients, underscoring the importance of improving study design and methodological rigor in future research.

## Supplementary Material

Manuscript_ID_BMB-2024-029_Supplement_ldaf004

## Data Availability

The data underlying this article are available in the article and in its online supplementary material.
