# Supplementary material for: Extracorporeal shock wave therapy for erectile dysfunction: rethinking study design, implementation, and analysis
Source: Br Med Bull. 2025 May 19;154(1):ldaf004. doi: 10.1093/bmb/ldaf004 (PMC12086999; doi:10.1093/bmb/ldaf004)
Supplement: Manuscript_ID_BMB-2024-029_Supplement_ldaf004 [file manuscript_id_bmb-2024-029_supplement_ldaf004.docx]

Extracorporeal shock wave therapy for erectile dysfunction: proposal for a fundamental change of mind in the planning, implementation and analysis of related clinical studies

Janak Desai, Eric Huyghe, Gayle D. Maffulli,

Carmen Nussbaum-Krammer, Jessica Tittelmeier, Christoph Schmitz

Supplementary Data

This Online Supplement contains the raw data of 55 variables extracted from 87 clinical trials on extracorporeal shock wave therapy for erectile dysfunction published until September 27, 2024.

**Abbreviations used in Supplementary Data:**

| 3D | Three-dimensional |
| --- | --- |
| BOCF | Baseline observation carried forward |
| C | Control treatment |
| D | Day |
| ED^+^ | Positive energy density |
| ED^total^ | Total energy density |
| EH | Electrohydraulic |
| EM | Electromagnetic |
| EM-B | Electromagnetic-ballistic |
| ESW | Extracorporeal shock wave |
| ESWT | Extracorporeal shock wave therapy |
| F | Focused |
| Hz | Hertz |
| IIEF-EF | International Index of Erectile Function - Erectile Domain |
| ITT | Intent-to-treat |
| KL | Kilovolt |
| L | Linear |
| mJ/mm^2^ | Millijoule per squared millimeter |
| n.a. | Not applicable |
| n.s. | Not specified |
| P-B | Pneumatic-ballistic |
| PDE5i | Phosphodiesterase-5 inhibitors |
| PE | Piezoelectric |
| PRP | Platelet derived plasma |
| R | Radial |
| Ref | Reference |
| RCT | Randomized controlled trial |
| SD | Standard deviation |
| TMS | Transcranial magnetic stimulation |
| U | Unfocused |
| V | Variable |
| W | Week |

| **Name** | **Variable** |
| --- | --- |
| **A - General information** | |
| V1 | Name of the first author |
| V2 | Year of publication |
| V3 | The country (or countries) in which the clinical trial was performed were specified (country name(s) / not specified) |
| V4 | The type of investigated erectile dysfunction was specified (vasculogenic / organic / post-radical prostatectomy / diverse / not specified) |
| V5 | The patients were non-responders to PDE5i (yes / no / both / not specified) |
| V6 | The blood testosterone level of all patients enrolled in the trial was normal (yes / no / not specified) |
| V7 | Testosterone replacement therapy was performed in case of testosterone deficiency (yes / no / not applicable) |
| V8 | The IIEF-EF score was determined to enable comparison of treatment outcome with other studies on ESWT for ED, including the first description in the literature (yes / no) |
| V9 | The type of clinical trial was specified (case series / cohort study / RCT) |
| V10 | Control groups were specified (not applicable / 1 / 2 / 3 / etc.) |
| V11 | It was specified whether the data were prospectively or retrospectively collected (prospective / retrospective / not specified) |
| V12 | The time post-baseline or post-treatment [weeks] at which the primary endpoint was determined was specified. (Note: in this systematic review sometimes data from a different time point were used for analysis in order to keep the times post-treatment as homogeneous as possible.) |
| V13 | Additional follow-up times [weeks or months] were reported (values / not applicable) |
| **B – ESWT device used** | |
| V14 | The exact name of the used device was specified (name / not specified) |
| V15 | The manufacturer's name, country and city of the headquarter were specified (data / not specified) |
| V16 | The type of generated ESWs was specified (focused (F) / unfocused (U) / linear (L) / radial (R) / not specified) |
| V17 | The technology of ESW generation was specified (electrohydraulic (EH) / electromagnetic (EM) / piezoelectric (PE) / electromagnetic-ballistic (EM-B) / pneumatic-ballistic (P-B) / not specified) |
| **C – (Main) ESWT group** | |
| V18 | The number of patients in the (main) ESWT group was specified (number / not specified) |
| V19 | The number of patients in the (main) ESWT group lost to follow-up was specified (number / not specified) |
| V20 | The age of the patients [years] in the (main) ESWT group was specified (minimum / median / mean / standard deviation / maximum / interquartile distance / not specified) |
| V21 | The duration of erectile dysfunction before baseline [months] of the patients in the (main) ESWT group was specified (minimum / median / mean / standard deviation / maximum / interquartile distance / not specified) |
| V22 | The number of treatment sessions was specified (number / not specified) |
| V23 | The time sequence of the treatment sessions was specified (weeks and number of sessions per week / not specified) |
| V24 | The number of ESWs per treatment session was specified (number of treatment regions distributed over the penis times number of ESWs per treatment region / not specified) |
| V25 | The type of the reported energy density of the ESWs was specified (ED^+^ / ED^total^ / not specified whether ED^+^ or ED^total^) |
| V26 | The energy density of the ESWs [mJ/mm^2^] was specified (value / not specified). |
| V27 | Alternatively, there was another description of the energy settings of ESWs (KV / mJ / Bar) |
| V28 | The three-dimensional (3D) acoustic pressure field of the applied ESWs (i.e., for the device settings used in the clinical trial) was analyzed, including determination of the 3D regional distribution of the peak positive pressure, peak negative pressure and the resulting energy density (yes / no) |
| V29 | Alternatively, the report referred to a publication in which the 3D acoustic pressure field of the applied ESWs (i.e., for the device settings used in the clinical trial) was analyzed as described in V28 (yes / no) |
| V30 | The frequency of the applied ESWs [Hz] was specified (value / not specified) |
| V31 | Additional treatment(s) next to ESWT were specified (additional treatment(s) / no additional treatment(s)) |
| **D – First control group (if applicable)** | |
| V32 | Data corresponding to V18 |
| V33 | Data corresponding to V19 |
| V34 | Data corresponding to V20 |
| V35 | Data corresponding to V21 |
| V36 | The treatment(s) of the patients in the first control group were specified (treatment(s) / not specified) |
| **E – Second control group (if applicable)** | |
| V37 | Data corresponding to V18 |
| V38 | Data corresponding to V19 |
| V39 | Data corresponding to V20 |
| V40 | Data corresponding to V21 |
| V41 | Data corresponding to V36 |
| **F – Third control group (if applicable)** | |
| V42 | Data corresponding to V18 |
| V43 | Data corresponding to V19 |
| V44 | Data corresponding to V20 |
| V45 | Data corresponding to V21 |
| V46 | Data corresponding to V36 |
| **G – Statistical analysis** | |
| V47 | An Intent-to-Treat analysis was performed (yes / no) |
| V48 | There were missing data due to patients lost to follow-up (yes / no) |
| V49 | Missing data imputation was performed (yes / no / not applicable as no patient was lost to follow-up) |
| V50 | The method(s) used for missing data imputation were specified (method(s) / not applicable) |
| V51 | An estimand strategy for handling intercurrent events was developed (yes / no) |
| V52 | The reported data are suitable for calculating an average mean ± SD of mean IIEF-EF data reported in different clinical trials at baseline and at follow-up (i.e., mean IIEF-EF values at baseline and at follow-up must be reported) (data) |
| V53 | The data were suitable for a meta-analysis (i.e., mean, SD and number of patients in the ESWT group and the main control group were available at baseline and at follow-up, and use or non-use of ESWT was the only difference between the groups (sham treatment was considered non-use of ESWT)) (yes / no / not applicable for case series) |
| **H – Therapeutic outcome** | |
| V54 | ESWT resulted in a statistically significant improvement of erectile dysfunction compared to baseline (yes / no / not applicable) |
| V55 | ESWT resulted in a statistically significant improvement of erectile dysfunction compared to sham or control treatment (not applicable for case series; A, ESWT+, C-, ESWT > C; B, ESWT+, C+, ESWT > C; C, ESWT+, C+, ESWT = C; D, ESWT-, C-, ESWT = C; E, ESWT-, C+, ESWT < C |

| **Ref** | **V1** | **V2** | **V3** | **V4** | **V5** | **V6** | **V7** | **V8** |
| --- | --- | --- | --- | --- | --- | --- | --- | --- |
| 1 | İbis | 2024 | Turkey | Not specified | Yes | Yes | Not applicable | No |
| 2 | Kalyvianakis | 2024 | Greece | Vasculogenic | No | Yes | Not applicable | Yes |
| 3 | Kaynak | 2024 | Turkey | Organic | Both | Not tested | Not considered | No |
| 4 | Rubino | 2024 | Italy | Vasculogenic | No | Yes | Not applicable | No |
| 5 | Trishch | 2024 | Ukraine | Not specified | No | Not tested | Not considered | Yes |
| 6 | Bayraktar | 2023 | Turkey | Not specified | Yes | Yes | Not applicable | No |
| 7 | Bryk | 2023 | USA | P-RP | No | Not tested | Not considered | No |
| 8 | Islam | 2023 | Bangladesh | Not specified | No | Not tested | Not considered | No |
| 9 | Jang | 2023 | Korea | P-RP | No | Not tested | Not considered | No |
| 10 | Kennady | 2023 | USA | Organic | Both | Yes | Not applicable | No |
| 11 | Kohada | 2023 | Japan | P-RP | No | Not tested | Not considered | Yes |
| 12 | Kurosawa | 2023 | Japan | Diverse | No | Yes | Not applicable | No |
| 13 | Ma | 2023 | China | Organic | No | Not tested | Not considered | No |
| 14 | Vena | 2023 | Italy | Organic | Yes | Yes | Not applicable | Yes |
| 15 | Wang | 2023 | China | Diverse | No | Not tested | Not considered | Yes |
| 16 | Cocci | 2022 | Italy | **^[[1]](#footnote-1)^** | No | Yes | Not applicable | No |
| 17 | Daneshwar | 2022 | Malaysia | **^[[2]](#footnote-2)^** | No | Not tested | Not considered | No |
| 18 | Ergün | 2022 | Turkey | Vasculogenic | Yes | Not tested | Not considered | No |
| 19 | Gallo | 2022 | Italy | Not specified | No | Yes | Not applicable | Yes |
| 20 | Geyik | 2022 | Turkey | Not specified | No | Yes | Not applicable | Yes |
| 21 | Kalyvianakis | 2022 | Greece | Vasculogenic | No | Yes | Not applicable | Yes |
| 22 | Motil | 2022 | Czech republic | P-RP | No | Yes | Not applicable | No |
| 23 | Mykoniatis | 2022 | Greece | Vasculogenic | No | Yes | Not applicable | Yes |
| 24 | Oginski | 2022 | Germany | Organic | Yes | Not tested | Not considered | No |
| 25 | Ong | 2022 | Malaysia | Diverse | No | Not tested | Not considered | No |
| 26 | Sandoval-Salinas | 2022 | Colombia | Organic | No | Not tested | Not considered | Yes |
| 27 | Scroppo | 2022 | Italy | Vasculogenic | Yes | Yes | Not applicable | No |
| 28 | Tao | 2022 | China | Vasculogenic | Yes | Yes | Not applicable | Yes |
| 29 | Zanaty | 2022 | Egypt | Organic | No | Yes | Not applicable | No |
| 30 | Adeldaeim | 2021 | Egypt | Vasculogenic | No | No | Yes | No |
| 31 | Akande | 2021 | Nigeria | Vasculogenic | No | Not tested | Not considered | No |
| 32 | Caretta | 2021 | Italy | Organic | No | Yes | Not applicable | No |
| 33 | Chung (follow-up) | 2021 | Australia | Vasculogenic | No | Not tested | Not considered | No |
| 34 | Geyik | 2021 | Turkey | Diverse | Yes | Yes | Not applicable | Yes |
| 35 | Karakose | 2021 | Turkey | P-RP | No | Not tested | Not considered | No |
| 36 | Ladegaard | 2021 | Denmark | P-RP | No | Not tested | Not considered | No |
| 37 | Lau | 2021 | Singapore | Diverse | No | Yes | Not applicable | No |
| 38 | Lei | 2021 | China | Diverse | No | Not tested | Not considered | No |
| 39 | Ortac | 2021 | Turkey | Vasculogenic | No | Yes | Not applicable | Yes |
| 40 | Palmieri | 2021 | Italy | Vasculogenic | Yes | Not tested | Not considered | Yes |
| 41 | Shendy | 2021 | Egypt | Vasculogenic | No | Not tested | Not considered | Yes |
| 42 | Tzou | 2021 | Taiwan | Diverse | Yes | Yes | Not applicable | No |
| 43 | Vinay | 2021 | Spain | Diverse | Yes | Not tested | Not considered | Yes |
| 44 | Baccaglini | 2020 | Brazil | P-RP | No | Not tested | Not considered | No |
| 45 | Eryilmaz | 2020 | Turkey | Vasculogenic | Yes | Yes | Not applicable | No |
| 46 | Huang | 2020 | China | Diverse | No | Yes | Not applicable | No |
| 47 | Kalyvianakis | 2020 | Greece | Vasculogenic | No | Not tested | Not considered | Yes |
| 48 | Konchugova | 2020 | Russia | Vasculogenic | No | Not tested | Not considered | No |
| 49 | Lurz | 2020 | USA | Vasculogenic | No | Yes | Not applicable | Yes |
| 50 | Inoue | 2020 | Japan | P-RP | No | Yes | Not applicable | No |
| 51 | Kim | 2020 | Korea | Not specified | No | Not tested | Not considered | Yes |
| 52 | Patel | 2020 | USA | Organic | No | Yes | Not applicable | Yes |
| 53 | Verze | 2020 | Italy | Vasculogenic | No | Not tested | Not considered | No |
| 54 | Wang | 2020 | China | Organic | Both | Yes | Not applicable | Yes |
| 55 | Wu | 2020 | USA | Vasculogenic | No | Not tested | Not considered | No |
| 56 | Zasieda | 2020 | Ukraine | **^[[3]](#footnote-3)^** | No | Yes | Not applicable | No |
| 57 | Costa | 2019 | Portugal | Organic | No | Not tested | Not considered | No |
| 58 | Musa | 2019 | Egypt | Vasculogenic | Yes | No | Yes | **^[[4]](#footnote-4)^** |
| 59 | Sramkova | 2019 | Czech republic | Vasculogenic | No | Yes | Not applicable | No |
| 60 | Vita | 2019 | Italy | Vasculogenic | Yes | Yes | Not applicable | No |
| 61 | Wang | 2019 | Taiwan | **^[[5]](#footnote-5)^** | Yes | Not tested | Not considered | No |
| 62 | Yamaçake | 2019 | Brazil | **^[[6]](#footnote-6)^** | No | Yes | Not applicable | No |
| 63 | De Oliveira | 2018 | Portugal | Diverse | No | Not tested | Not considered | No |
| 64 | Fojecki | 2018 | Demnark | Organic | No | Yes | Not applicable | Yes |
| 65 | Kalyvianakis | 2018 | Greece | Vasculogenic | No | Not tested | Not considered | Yes |
| 66 | Kitrey | 2018 | Israel | Diverse | No | Not tested | Not considered | **^4^** |
| 67 | Zewin | 2018 | Egypt | P-RP | No | Not tested | Not considered | No |
| 68 | Ayala | 2017 | Colombia / Spain | Vasculogenic | No | Not tested | Not considered | No |
| 69 | Fojecki | 2017 | Denmark | Organic | No | Yes | Not applicable | Yes |
| 70 | Kalyvianakis | 2017 | Greece | Vasculogenic | No | Not tested | Not considered | Yes |
| 71 | Tsai | 2017 | Taiwan | Organic | Yes | No | Yes | No |
| 72 | Bechara | 2016 | Argentina | Vasculogenic | Yes | Not tested | Not considered | **^[[7]](#footnote-7)^** |
| 73 | Frey | 2016 | Denmark | P-RP | No | Not tested | Not considered | No |
| 74 | Hisasue | 2016 | Japan | Diverse | No | Yes | Not applicable | No |
| 75 | Kitrey | 2016 | Israel | Vasculogenic | Yes | Not tested | Not considered | Yes |
| 76 | Bechara | 2015 | Argentina | Vasculogenic | Yes | Not tested | Not considered | Yes |
| 77 | Chung | 2015 | Australia | Vasculogenic | No | Not tested | Not considered | No |
| 78 | Olsen | 2015 | Denmark | Organic | No | Not tested | Not considered | No |
| 79 | Pelayo-Nieto | 2015 | Mexico | Vasculogenic | No | Not tested | Not considered | Yes |
| 80 | Reisman | 2015 | **^[[8]](#footnote-8)^** | Vasculogenic | No | Not tested | Not considered | Yes |
| 81 | Ruffo | 2015 | Italy | Organic | Yes | Not tested | Not considered | Yes |
| 82 | Srini | 2015 | India | Organic | No | Not tested | Not considered | Yes |
| 83 | Yee | 2014 | China | Organic | No | Not tested | Not considered | Yes |
| 84 | Gruenwald | 2012 | Israel | Vasculogenic | No | Not tested | Not considered | Yes |
| 85 | Palmieri | 2012 | Italy | ED + PD | No | Not tested | Not considered | No |
| 86 | Vardi | 2012 | Israel | Vasculogenic | No | Not tested | Not considered | Yes |
| 87 | Vardi | 2010 | Israel | Vasculogenic | No | Not tested | Not considered | Yes |

| **Ref** | **V9** | **V10** | **V11** | **V12** | **V13** | **V14** | **V15** | **V16** | **V17** |
| --- | --- | --- | --- | --- | --- | --- | --- | --- | --- |
| 1 | Cohort study | 2 | Retrospective | 27 |  | Duolith SD1 | Storz Medical | F | EM |
| 2 | RCT | 1 | Prospective | 16 |  | Aries 2 | Dornier | F | EM |
| 3 | Case series | n.a. | Retrospective | 52 | 208 | Omnispec ED1000 | Medispec | F | EH |
| 4 | Case series | n.a. | Retrospective | 11 | 52 | Ortho Gold 100 | MTS | F | EH |
| 5 | Cohort study | 2 | Not specified | 8 |  | GentlePro | Zimmer | R | R |
| 6 | Case series**^[[9]](#footnote-9)^** | n.a. | Retrospective | 21 | 52 | Not specified | Unknown | n.s. | n.s. |
| 7 | Cohort study | 1 | Prospective | 12 |  | enPulse Pro | Zimmer | R | R |
| 8 | Case series | n.a. | Prospective | 7 | 52 | BTL-6000 | BTL | R | R |
| 9 | Cohort study | 1 | Prospective | 24 |  | Omnispec ED1000 | Medispec | F | EH |
| 10 | RCT | 1 | Prospective | 4 |  | Duolith SD1 | Storz Medical | F | EM |
| 11 | Cohort study | 1 | Retrospective | 24 |  | Omnispec ED1000 | Medispec | F | EH |
| 12 | Cohort study | 1 | Retrospective | 8 |  | Omnispec ED1000 | Medispec | F | EH |
| 13 | Case series | n.a. | Not specified | 24 | 52 | LGT-2510B | Longest | n.s. | n.s. |
| 14 | Case series | n.a. | Prospective | 30 |  | UroGold 100 | MTS | F | EH |
| 15 | Cohort study | 1 | Prospective | 13 |  | Omnispec ED1000 | Medispec | F | EH |
| 16 | Case series | n.a. | Prospective | 20 |  | Duolith SD1 | Storz Medical | F | EM |
| 17 | Case series | n.a. | Prospective | 10 |  | Duolith SD1 | Storz Medical | F | EM |
| 18 | Case series | n.a. | Retrospective | 12 |  | Not specified | Electronica Pagani | n.s. | n.s. |
| 19 | RCT | 1 | Prospective | 24 | 52 | Duolith SD1 | Storz Medical | F | EM |
| 20 | Case series | n.a. | Retrospective | 24 |  | Renova | Direx | L | EM |
| 21 | RCT | 1 | Prospective | 12 |  | Aries 2 | Dornier | F | EM |
| 22 | RCT | 1 | Prospective | 24 |  | Piezowave 2 linear | Richard Wolf | L | PE |
| 23 | RCT | 1 | Prospective | 28 |  | Aries 2 | Dornier | F | EM |
| 24 | Case series | n.a. | Prospective | 30 |  | Duolith SD1 | Storz Medical | F | EM |
| 25 | RCT | 1 | Prospective | 24 |  | Piezowave 2 linear | Richard Wolf | L | PE |
| 26 | RCT | 1 | Prospective | 10 |  | Masterpuls MP50 | Storz Medical | R | R |
| 27 | Case series | n.a. | Prospective | n.s. |  | Duolith SD1 | Storz Medical | F | EM |
| 28 | RCT | 2 | Prospective | 12 |  | ESWO-I 80 mm | SHME**^[[10]](#footnote-10)^** | F | EM |
| 29 | Cohort study | 1 | Prospective | 12 |  | Piezowave 2 linear | Richard Wolf | L | PE |
| 30 | Case series | n.a. | Prospective | 24 |  | Piezowave 2 focused | Richard Wolf | F | PE |
| 31 | Case series | n.a. | Prospective | 24 |  | Omnispec ED1000 | Medispec | F | EH |
| 32 | Case series | n.a. | Retrospective | 24 |  | Duolith SD1 | Storz Medical | F | EM |
| 33 | Case series | n.a. | Prospective | 260 |  | Duolith SD1 | Storz Medical | F | EM |
| 34 | Cohort study | 1 | Retrospective | 24 |  | Renova | Direx | L | EM |
| 35 | Cohort study | 1 | Retrospective | 52 |  | Omnispec ED1000 | Medispec | F | EH |
| 36 | RCT | 1 | Prospective | 12 |  | Duolith SD1 | Storz Medical | F | EM |
| 37 | Case series | n.a. | Prospective | 24 |  | Duolith SD1 | Storz Medical | F | EM |
| 38 | Cohort study | 1 | Prospective | 12 |  | Omnispec ED1000 | Medispec | F | EH |
| 39 | RCT | 1 | Prospective | 24 |  | Duolith SD1 | Storz Medical | F | EM |
| 40 | Case series | n.a. | Prospective | 7 |  | Duolith SD1 | Storz Medical | F | EM |
| 41 | RCT | 1 | Prospective | 24 |  | Intellect**^[[11]](#footnote-11)^** | Chattanooga | F | EM |
| 42 | Case series | n.a. | Retrospective | 24 | 52 | Duolith SD1 | Storz Medical | F | EM |
| 43 | RCT | 1 | Prospective | 24 |  | Renova | Direx | L | EM |
| 44 | RCT | 1 | Prospective | 16 |  | Renova | Direx | L | EM |
| 45 | Cohort study | 1 | Not specified | 18 |  | Not specified | Electronica Pagani | F/U | n.s. |
| 46 | Case series | n.a. | Prospective | 8 |  | Renova | Direx | L | EM |
| 47 | RCT | 3 | Prospective | 24 |  | Aries 2 | Dornier | F | EM |
| 48 | RCT | 1 | Prospective | n.s. |  | Well Wave | Richard Wolf | F | PE |
| 49 | Case series | n.a. | Prospective | 10 |  | Piezowave 2 linear | Richard Wolf | L | PE |
| 50 | Cohort study | 2 | Not specified | 24 |  | Omnispec ED1000 | Medispec | F | EH |
| 51 | RCT | 1 | Prospective | 7 |  | MT 2000H | Urontech | F | EM |
| 52 | RCT | 1 | Prospective | 24 |  | MoreNova | Direx | L | EM |
| 53 | Cohort study | 1 | Retrospective | 31 |  | Omnispec ED1000 | Medispec | F | EH |
| 54 | Case series | n.a. | Not specified | 16 |  | HB-ESWT-01 | ZHME**^[[12]](#footnote-12)^** | F | n.s. |
| 55 | Cohort study | 1 | Retrospective | 12 |  | UroGold 100 | MTS | F | EH |
| 56 | RCT | 1 | Prospective | 24 |  | Duolith SD1 | Storz Medical | F | EM |
| 57 | Case series | n.a. | Not specified | 12 | 260 | Duolith SD1 | Storz Medical | F | EM |
| 58 | Case series | n.a. | Prospective | 24 | 76 | Aries 2 | Dornier | F | EM |
| 59 | RCT | 1 | Prospective | 12 |  | Piezowave 2 linear | Richard Wolf | L | PE |
| 60 | Case series | n.a. | Prospective | 18 |  | Omnispec ED1000 | Medispec | F | EH |
| 61 | Case series | n.a. | Prospective | 24 |  | Piezowave 2 linear | Richard Wolf | L | PE |
| 62 | RCT | 1 | Prospective | 12 |  | Swiss DolorClast | EMS | R | R |
| 63 | Case series | n.a. | Prospective | 18 |  | Piezowave 2 linear | Richard Wolf | L | PE |
| 64 | RCT | 1 | Prospective | 24 | 52 | Piezowave 2 linear | Richard Wolf | L | PE |
| 65 | RCT | 1 | Prospective | 24 |  | Aries 2 | Dornier | F | EM |
| 66 | Case series | n.a. | Prospective | 24 | 108 | Not specified | Unknown | n.s. | n.s. |
| 67 | RCT | 2 | Prospective | 24 | 36 | Aries 2 | Dornier | F | EM |
| 68 | Case series | n.a. | Retrospective | 36 |  | Duolith SD1 | Storz Medical | F | EM |
| 69 | RCT | 1 | Prospective | 9 |  | Piezowave 2 linear | Richard Wolf | L | PE |
| 70 | RCT | 1 | Prospective | 24 | 52 | Omnispec ED1000 | Medispec | F | EH |
| 71 | Case series | n.a. | Prospective | 24 |  | Duolith SD1 | Storz Medical | F | EM |
| 72 | Case series | n.a. | Prospective | 24 |  | Renova | Direx | L | EM |
| 73 | Case series | n.a. | Not specified | 10 |  | Duolith SD1 | Storz Medical | F | EM |
| 74 | Case series | n.a. | Prospective | 24 |  | Omnispec ED1000 | Medispec | F | EH |
| 75 | RCT | 1 | Prospective | 13 |  | Omnispec ED1000 | Medispec | F | EH |
| 76 | Case series | n.a. | Prospective | 24 |  | Renova | Direx | L | EM |
| 77 | Case series | n.a. | Prospective | 20 |  | Duolith SD1 | Storz Medical | F | EM |
| 78 | RCT | 1 | Prospective | 5 |  | Duolith SD1 | Storz Medical | F | EM |
| 79 | Case series | n.a. | Not specified | 8 |  | Renova | Direx | L | EM |
| 80 | Case series | n.a. | Prospective | 24 |  | Renova | Direx | L | EM |
| 81 | Case series | n.a. | Not specified | 12 |  | Renova | Direx | L | EM |
| 82 | RCT | 1 | Prospective | 13 |  | Omnispec ED1000 | Medispec | F | EH |
| 83 | RCT | 1 | Prospective | 13 |  | Omnispec ED1000 | Medispec | F | EH |
| 84 | Case series | n.a. | Prospective | 13 |  | Not specified | Unknown | n.s. | n.s. |
| 85 | RCT | 1 | Prospective | 24 |  | Duolith SD1 | Storz Medical | F | EM |
| 86 | RCT | 1 | Prospective | 13 |  | Omnispec ED1000 | Medispec | F | EH |
| 87 | Case series | n.a. | Not specified | 24 |  | Omnispec ED1000 | Medispec | F | EH |

| **Ref** | **V18** | **V19** | **V20^[[13]](#footnote-13)^** | **V20^[[14]](#footnote-14)^** | **V20^[[15]](#footnote-15)^** | **V20^[[16]](#footnote-16)^** | **V21113** | **V21^14^** | **V2115** | **V21^[[17]](#footnote-17)^** |
| --- | --- | --- | --- | --- | --- | --- | --- | --- | --- | --- |
| 1 | 36 | 0 | 56.1 |  | 7.3 |  |  |  |  | >6 |
| 2 | 34 | 0 | 59 |  | 8.6 |  | 94 |  | 75 |  |
| 3 | 116 | 0 | 47.3 |  |  | 19-71 |  |  |  | <6 |
| 4 | 113 | 0 |  | 50 |  |  | n.s. |  |  |  |
| 5 | 34 | 0 | 35.3 |  |  | 25-50 | 23.4 |  |  | 6-84 |
| 6 | 126 | 0 | 50.5 |  | 12.4 |  |  |  |  | >6 |
| 7 | 44 | 1 | 59.5 |  | 6.9 |  | n.a. |  |  |  |
| 8 | 31 | 0 | 44.6 |  |  | 25-78 |  |  |  | >6 |
| 9 | 56 | 15 | 62 |  |  |  | n.a. |  |  |  |
| 10 | 17 | 0 | 67 |  |  |  | n.s. |  |  |  |
| 11 | 16 | **^[[18]](#footnote-18)^** | 63 |  |  | 52-72 | n.a. |  |  |  |
| 12 | 76 | 0 | 52.8 |  | 11.7 |  | n.s. |  |  |  |
| 13 | 26 | 0 | 43.8 |  | 8.1 |  | n.s. |  |  |  |
| 14 | 21 | 3 | 56.3 |  | 9.1 |  | >12 |  |  |  |
| 15 | 51 | 9 | 33.9 |  | 6.2 |  | 24 |  |  | 12-36 |
| 16 | 5 | 0 | 31.2 |  |  | 24-36 | n.s. |  |  |  |
| 17 | 50 | 0 | 41.9 |  | 11.7 |  | n.s. |  |  |  |
| 18 | 63 | 0 | 57.1 |  | 5.1 |  | 25 |  | 5.5 |  |
| 19 | 50 | 9 | 50.5 |  | 13.7 |  |  |  |  | >3 |
| 20 | 41 | 0 | 51.6 |  | 11.8 |  | 54.5 |  | 33 |  |
| 21 | 35 | 1 |  | 54 |  |  |  | 68 |  |  |
| 22 | 20 | 4 | 58.3 |  | 4.9 |  |  | 7 |  | 3-18 |
| 23 | 25 | 0 |  | 56 |  |  |  | 44 |  |  |
| 24 | 50 | 0 |  | 59 |  |  |  | 48 |  |  |
| 25 | 27 | 0 |  | 61 |  |  |  | 24 |  |  |
| 26 | 40 | 3 | 47.7 |  | 12.8 |  | 5.5 |  | 6.6 |  |
| 27 | 30 | 0 | 52.8 |  | 7.9 |  | n.s. |  |  |  |
| 28 | 35 | 2 | 48.3 |  | 3.5 |  | 45.3 |  | 25.1 |  |
| 29 | 25 | 0 | 43.8 |  |  | 28-62 | 66 |  |  | 12-180 |
| 30 | 425 | 0 |  | 55 |  | 30-70 | 25 |  |  | 12-38 |
| 31 | 30 | 8 | 53.7 |  | 8 |  |  |  |  | >6 |
| 32 | 111 | 0 | 53.7 |  | 11.6 |  | n.s. |  |  |  |
| 33 | 30 | 0 | 55.8 |  |  | 42-68 | 21.8 |  |  | 6-60 |
| 34 | 91 | 0 | 46.9 |  | 11.9 |  | 48 |  | 32 |  |
| 35 | 34 | 0 | 59.2 |  |  | 51-70 | n.a. |  | -- |  |
| 36 | 20 | 0 | 60.8 |  | 6.8 |  | 11.2 |  | 4.5 |  |
| 37 | 19 | 8 | 57.4 |  |  | 38-70 | 46.3 |  |  | 12-120 |
| 38 | 60 | 14 | 33.4 |  | 6.2 |  | 24 |  |  | 12-36 |
| 39 | 44 | 25 | 42.3 |  | 9.9 |  | 33.7 |  | 36.5 |  |
| 40 | 109 | 3 | 57.9 |  |  | 21-78 | 33.3 |  |  | 3-103 |
| 41 | 21 | 0 | 48.6 |  | 5.5 |  | 118 |  | 13 |  |
| 42 | 69 | 0 |  | 55 |  | 45-66 |  | 12 |  | 6-36 |
| 43 | 40 | 0 |  | 60 |  | 54-66 |  | 36 |  | 24-72 |
| 44 | 46 | 10 | 64.6 |  | 5.3 |  | n.a. |  |  |  |
| 45 | 20 | 0 | 45.6 |  |  |  | n.s. |  |  |  |
| 46 | 35 | 0 | 33 |  |  | 21-68 |  |  |  | >6 |
| 47 | 24 | 1 | 58.7 |  | 9.5 |  |  |  |  | >6 |
| 48 | 20 | 0 | 59.2 |  | 8.7 |  | n.s. |  |  |  |
| 49 | 25 | 3 |  | 60 |  | 55-69 |  | 60 |  | 18-222 |
| 50 | 5 | **^[[19]](#footnote-19)^** | 62.2 |  | 2.7 |  | n.a. |  |  |  |
| 51 | 46 | 8 | 63.2 |  | 5.4 |  |  |  |  | >6 |
| 52 | 45 | 5 | 53.2 |  |  | 30-78 |  |  |  | >6 |
| 53 | 78 | 0 | 56 |  | 9.6 |  | n.s. |  |  |  |
| 54 | 45 | 0 |  | 60 |  | 30-84 |  | 38 |  | 6-120 |
| 55 | 24 | 0 |  | 61 |  | 48-70 |  | 61 |  | 36-61 |
| 56 | 22 | 0 | 51 |  |  | 45-60 | n.s. |  |  |  |
| 57 | 18 | 0 | 61.1 |  | 7.2 |  |  |  |  | >12 |
| 58 | 55 | 3 | 51 |  |  | 23-74 | 34.5 |  |  | 9-60 |
| 59 | 30 | 0 | 53.9 |  | 9.3 |  | 42 |  |  | 6-204 |
| 60 | 20 | 0 | 58.5 |  | 10.3 |  | 98.4 |  | 80.4 |  |
| 61 | 12 | 0 | 35.5 |  | 7.1 |  |  |  |  | >6 |
| 62 | 10 | 0 | 55.1 |  |  | 47-60 | 22.7 |  | 9.2 |  |
| 63 | 25 | 0 |  | 61 |  | 27-73 | 24 |  |  |  |
| 64 | 63 | 11 | 65.4 |  | 7.9 |  |  |  |  | >6 |
| 65 | 21 | 3 | 57.5 |  | 10 |  |  |  |  | >3 |
| 66 | 156 | 0 | 59.1 |  | 10.1 |  | 64.6 |  | 49 |  |
| 67 | 49 | 7 | 52.9 |  | 7.2 |  | n.a. |  |  |  |
| 68 | 710 | 298 |  | 58 |  | 24-83 |  |  |  | >3 |
| 69 | 63 | 3 |  | 65.4 | 7.9 |  |  |  |  | >6 |
| 70 | 30 | 0 |  | 53 |  | 31-72 |  | 66 |  | 12-240 |
| 71 | 52 | 0 | 60.1 |  | 11.5 |  | 38.3 |  | 26.9 |  |
| 72 | 25 | 5 | 64.8 |  |  | 48-82 | 70.5 |  | n.s. |  |
| 73 | 18 | 3 |  | 62 |  | 51-70 |  | 24 |  | 12-54 |
| 74 | 56 | 0 |  | 64 |  | 27-83 |  | 36 |  | 6-216 |
| 75 | 40 | 3 |  | 60 |  | 28-27 |  | 60 |  | 11-240 |
| 76 | 25 | 0 |  | 63 |  | 46-78 |  | 42 |  | 12-132 |
| 77 | 30 | 0 | 55.8 |  |  | 42-68 | 21.8 |  |  | 6-60 |
| 78 | 51 | 15 |  | 59 |  | 41-80 |  | 57 |  | 9-240 |
| 79 | 15 | 0 |  | 59.6 |  | 45-70 | n.s. |  |  |  |
| 80 | 58 | 0 | 56.8 |  |  | 33-84 |  |  |  | >3 |
| 81 | 31 | 0 | 59.9 |  | 12.2 |  |  |  |  | >6 |
| 82 | 95 | 35 | n.s. |  |  |  |  |  |  | >6 |
| 83 | 36 | 6 | 58.9 |  | 7.6 |  | 78 |  | 34 |  |
| 84 | 33 | 4 | 61.3 |  |  | 41-79 |  | 60 | n.s. |  |
| 85 | 50 | 0 | 55.5 |  |  | 32-75 | 8.8 |  |  | 6-12 |
| 86 | 46 | 6 |  | 58 |  | 27-72 |  | 42 |  | 6-240 |
| 87 | 20 | 0 | 56.1 |  |  | 33-73 | 34.7 |  | n.s. |  |

| **Ref** | **V22** | **V23** | **V24** | **V25** | **V26** | **V27** | **V28** | **V29** | **V30** |
| --- | --- | --- | --- | --- | --- | --- | --- | --- | --- |
| 1 | 6 | W1-W3 (2) | 6 x 500 | n.s. | 0.25 |  | No | No | n.s. |
| 2 | 12 | W1-W6 (2) | 2 x 2000 + 1 x 1000 | n.s. | 0.096 |  | No | No | 5 |
| 3 | 6 | W1-W3 (2) | 5 x 300 | n.s. | 0.09 |  | No | No | 2.66 |
| 4 | 8 | W1-W8 (1) | 5 x 300 | n.s. | n.s. |  | No | No | n.s. |
| 5 | 8 | W1-W8 (1) | 4 x 1500 | n.s. |  | 90-120 mJ**^[[20]](#footnote-20)^** | No | No | 12 |
| 6 | 12 | W2-W2 (3) and W5-W6 (3) | 3 x 500 | n.s. | 0.1 |  | No | No | 5 |
| 7 | 6 | W1-W6 (1) | 8 x 1250 | n.s. |  | 90 mJ**^20^** | No | No | 15 |
| 8 | 8 | W1-W3 (2 - 3) | 5 x 600 | n.s. |  | 1.2-2 bar**^[[21]](#footnote-21)^** | No | No | 7-12 |
| 9 | 6 | D4, D5, D6, D7, W2, W4 post-OP | 5 x 300 | n.s. | 0.09 |  | No | No | 2 |
| 10 | 6 | W1-W3 (2) | 2 x 1000 + 2 x 500 | n.s. | 0.1 |  | No | No | 5 |
| 11 | 12 | W1-W2 (3) and W3-W8 (1) | 5 x 300 | n.s. | 0.09 |  | No | No | 2 |
| 12 | 6 | W1-W3 and W7-W9 (1) | 3 x 1000 | n.s. | 0.09 |  | No | No | 5 |
| 13 | 6 | W1-W6 (1) | 2 x 1600 + 2 x 900 | n.s. | 0.09 |  | No | No | n.s. |
| 14 | 6 | W1-W6 (1) | 5 x 300 | n.s. | 0.09 |  | No | No | 8 |
| 15 | 12 | W1-W2 (3) and W7-W9 (2) | 5 x 300 | n.s. | 0.09 |  | No | No | 2 |
| 16 | 8 | D45 (1) and D52-D58 (1) | 2 x 1500 | n.s. | 0.25 |  | No | No | n.s. |
| 17 | 10 | W1-W5 (2) | 3000 (perineum) | n.s. | 0.25 |  | No | No | 5 |
| 18 | 12 | W1-W2 (3) and W7-W9 (2) | 10 x 500 | n.s. | 0.15 |  | No | No | 2 |
| 19 | 6 | W1-W6 (1) | 6 x 500 | n.s. | 0.25 |  | No | No | 4 |
| 20 | 10 | W1-W5 (1) and W25-W30 (1) | 2 x 1800 | n.s. | 0.09 |  | No | No | n.s. |
| 21 | 12 | W1-W6 (2) | 2 x 2000 + 1 x 1000 | n.s. | 0.096 |  | No | No | 5 |
| 22 | 4 | W1-W4 (1) | 2 x 2000 | n.s. | 0.16 |  | No | No | 8 |
| 23 | 6 | W1-W3 (2) | 2 x 2000 + 1 x 1000 | n.s. | 0.096 |  | No | No | 5 |
| 24 | 6 | W1-W6 (1) | 1 x 2000 + 1 x 1000 | n.s. | 0.2-0.25 |  | No | No | n.s. |
| 25 | 4 | W1-W4 (1) | 2 x 2000 | n.s. | n.s. |  | No | No | 8 |
| 26 | 6 | W1-W6 (1) | 1 x 4000 | n.s. |  | 2.4 bar**^21^** | No | No | 17 |
| 27 | 6 | W1, W3, W5 (2) | 2 x 1000 + 2 x 500 | n.s. | 0.25 |  | No | No | 3 |
| 28 | 12 | W1-W3 and W7-W9 (1) | 6 x 300 to 400 | n.s. |  | 7.5 KV**^[[22]](#footnote-22)^** | No | No | 1.66 |
| 29 | 6 | W1-W3 (2) | 2 x 3000 | n.s. | n.s. |  | No | No | n.s. |
| 30 | 6 | W1-W6 (1) | 5 x 1200 | n.s. | 0.2 |  | No | No | 8 |
| 31 | 12 | W1-W2 (3) and W7-W9 (2) | 5 x 300 | n.s. | n.s. |  | No | No | n.s. |
| 32 | 6 | W1-W6 (1) | 8 x 300 | n.s. | 0.12 |  | No | No | 3 |
| 33 | 12 | W1-W6 (2) | 2 x 1000 + 2 x 500 | n.s. | 0.25 |  | No | No | 6 |
| 34 | 5 | W1-W5 (1) | 2 x 1800 | n.s. | 0.09 |  | No | No | n.s. |
| 35 | 12 | W1-W6 (2) | 5 x 300 | n.s. | 0.09 |  | No | No | 2.66 |
| 36 | 5 | W1-W5 (1) | 6 x 500 | n.s. | 0.15 |  | No | No | 5 |
| 37 | 6**^[[23]](#footnote-23)^** | W1-W6 (1 or 2) | Not specified | n.s. | n.s. |  | No | No | n.s. |
| 38 | 12 | W1-W3 and W7-W9 (1) | 5 x 300 | n.s. | 0.09 |  | No | No | 2 |
| 39 | 4 | W1-W4 (1) | 10 x 300 | n.s. | 0.2 |  | No | No | 5 |
| 40 | 6 | W1-W3 (2) | 2 x 1000 + 2 x 500 | n.s. | 0.25 |  | No | No | 4-6 |
| 41 | 12 | W1-W3 (2) and W7-W9 (2) | 5 x 600 | n.s. | 0.09 |  | No | No | n.s. |
| 42 | 12 | W1-W12 (1) | 6 x 500 | n.s. | 0.2 |  | No | No | 4 |
| 43 | 4 | W1-W4 (1) | 2 x 1600 + 2 x 900 | n.s. | 0.09 |  | No | No | 2 |
| 44 | 8 | W1-W8 (1) | 4 x 600 | n.s. | 0.09 |  | No | No | 5 |
| 45 | 12 | W1-W3 and W7-W9 (1) | 6 x 300 | n.s. | 0.15 |  | No | No | n.s. |
| 46 | 4 | W1-W4 (1) | 2 x 1600 + 2 x 900 | n.s. | 0.09 |  | No | No | 5 |
| 47 | 12 | W1-W6 (2) | 4 x 1000 | n.s. | 0.05 |  | No | No | 8 |
| 48 | 7 | W1-W3 (2 to 3) | 1 x 1500 | n.s. | n.s. |  | No | No | 4 |
| 49 | 6 | W1-W6 (1) | 2 x 3300 | n.s. | 0.16 |  | No | No | n.s. |
| 50 | 12 | W1-W2 (3) and W3-W8 (1) | 5 x 300 | n.s. | 0.09 |  | No | No | 2 |
| 51 | 12 | W1-W3 and W7-W9 (1) | 3 x 1000 | n.s. | **^[[24]](#footnote-24)^** |  | No | No | 5 |
| 52 | 5 | D1-D5 (1 per day) | 2 x 360 | n.s. | n.s. |  | No | No | n.s. |
| 53 | 6 | W1-W3 (2) | 1500 - 2400 | n.s. | 0.09 |  | No | No | 2 |
| 54 | 8 | W1-W4 (2) | 4 x 1600 + 3 x 1200 | n.s. | 0.05 |  | No | No | 3.33 |
| 55 | 6 | W1-W6 (1) | 1 x 3000 | n.s. | 0.09 |  | No | No | n.s. |
| 56 | 12 | W1-W6 (2) | 1 x 3000 | n.s. | 0.25 |  | No | No | 3 |
| 57 | 4 | W1-W4 (1) | 5 x 600 | n.s. | 0.15 |  | No | No | 5 |
| 58 | 12 | W1-W3 and W7-W9 (1) | 5 x 300 | n.s. | 0.009 |  | No | No | n.s. |
| 59 | 4 | W1-W2 (2) | 2 x 3000 | n.s. | 0.16 |  | No | No | n.s. |
| 60 | 6**^[[25]](#footnote-25)^** | W1-W3 (2) | 5 x 300 | n.s. | 0.09 |  | No | No | 2 |
| 61 | 6 | W1-W6 (1) | 2 x 1000 | n.s. | 0.16 |  | No | No | n.s. |
| 62 | 6 | W1-W3 (2) | 1 x 2000 | n.s. | 0.09 |  | No | No | n.s. |
| 63 | 6 | W1-W6 (1) | 2 x 2000 | n.s. | 0.16 |  | No | No | n.s. |
| 64 | 10 | W1-W5 (1) and W10-W14 (1) | 1 x 600 | n.s. | 0.09 |  | No | No | 5 |
| 65 | 18 | W1-W6 (1) and W25-W30 (2) | 4 x 1000 + 2 x 500 | n.s. | 0.05 |  | No | No | 8 |
| 66 | 12 | W1-W3 and W7-W9 (1) | 5 x 300 | n.s. | 0.09 |  | No | No | 2 |
| 67 | 12 | W1-W3 and W7-W9 (1) | 5 x 300 | n.s. | 0.09 |  | No | No | 2 |
| 68 | 5 | W1-W5 (1) | 6 x 500 | n.s. | 0.1 |  | No | No | n.s. |
| 69 | 10 | W1-W5 (1) and W10-W14 (1) | 1 x 600 | n.s. | 0.09 |  | No | No | 5 |
| 70 | 12 | W1-W3 and W7-W9 (1) | 5 x 300 | n.s. | 0.09 |  | No | No | 2.66 |
| 71 | 12 | W1-W12 (1) | 6 x 500 | n.s. | 0.15 |  | No | No | 4 |
| 72 | 4 | W1-W4 (1) | 4 x 900 | n.s. | 0.09 |  | No | No | n.s. |
| 73 | 6 | W1-W6 (1) | 3 x 1000 | n.s. | **^24^** |  | No | No | 5 |
| 74 | 12 | W1-W3 and W7-W9 (1) | 5 x 300 | n.s. | 0.09 |  | No | No | 2 |
| 75 | 12 | W1-W3 and W7-W9 (1) | 5 x 300 | n.s. | 0.09 |  | No | No | 2 |
| 76 | 4 | W1-W4 (1) | 2 x 1600 + 2 x 900 | n.s. | 0.09 |  | No | No | n.s. |
| 77 | 12 | W1 (2) to W6 (2) | 2 x 1000 + 2 x 500 | n.s. | 0.25 |  | No | No | 6 |
| 78 | 5 | W1-W5 (1) | 6 x 500 | n.s. | 0.15 |  | No | No | 5 |
| 79 | 4 | W1-W4 (1) | 2x 1600 + 2 x 900 | n.s. | 0.09 |  | No | No | 5 |
| 80 | 4 | W1-W4 (1) | 4 x 900 | n.s. | 0.09 |  | No | No | 5 |
| 81 | 4 | W1-W4 (1) | 4 x 900 | n.s. | 0.09 |  | No | No | n.s. |
| 82 | 12 | W1-W3 and W7-W9 (1) | 5 x 300 | n.s. | 0.09 |  | No | No | 2 |
| 83 | 12 | W1-W3 and W7-W9 (1) | 5 x 300 | n.s. | 0.09 |  | No | No | 2 |
| 84 | 12 | W1-W3 and W7-W9 (1) | 5 x 300 | n.s. | 0.09 |  | No | No | 2 |
| 85 | 4 | W1-W4 (1) | 2000 | n.s. | 0.25 |  | No | No | 4 |
| 86 | 12 | W1-W3 and W7-W9 (1) | 5 x 300 | n.s. | 0.09 |  | No | No | 2 |
| 87 | 12 | W1-W3 and W7-W9 (1) | 5 x 300 | n.s. | 0.09 |  | No | No | 2 |

| **Ref** | **V31** |
| --- | --- |
| 1 | PDE5i (20 mg of tadalafil every second day) |
| 2 | PDE5i (daily from W1-W4) |
| 3 | PDE5i (daily for 6 months) |
| 4 | -- |
| 5 | PDE5i + L-arginine |
| 6 | PDE5i (daily for 2 months) |
| 7 | -- |
| 8 | -- |
| 9 | PDE5i (daily for 6 months post-OP) |
| 10 | -- |
| 11 | -- |
| 12 | -- |
| 13 | -- |
| 14 | PDE5i (daily starting at W11) |
| 15 | --- |
| 16 | -- |
| 17 | -- |
| 18 | PDE5i (daily) |
| 19 | PDE5i + L-arginine |
| 20 | -- |
| 21 | -- |
| 22 | -- |
| 23 | PDE5i (daily from W1-W4) |
| 24 | -- |
| 25 | -- |
| 26 | 2000 shots into bilateral ischiocavernosus and bulbospongiosus muscles |
| 27 | -- |
| 28 | Vacuum erectile device (3x /week; 9 weeks) |
| 29 | -- |
| 30 | -- |
| 31 | -- |
| 32 | -- |
| 33 | -- |
| 34 | PRP (3x, W1, W3, W5) |
| 35 | PDE5i (daily) |
| 36 | -- |
| 37 | -- |
| 38 | -- |
| 39 | -- |
| 40 | -- |
| 41 | Kegel exercises three times daily for 6 weeks |
| 42 | -- |
| 43 | -- |
| 44 | PDE5i (daily for8 weeks post-OP) |
| 45 | -- |
| 46 | -- |
| 47 | -- |
| 48 | TMS |
| 49 | -- |
| 50 | -- |
| 51 | -- |
| 52 | -- |
| 53 | PDE5i (daily for 12 weeks) |
| 54 | PDE5i (daily or on demand) |
| 55 | PDE5i (on demand) |
| 56 | Chorionic gonadotropin + extract of Epimedium Breviconum |
| 57 | -- |
| 58 | -- |
| 59 | -- |
| 60 | PDE5i (daily or on demand) |
| 61 | PDE5i (daily for 6 months post-accident) |
| 62 | -- |
| 63 | PDE5i (daily for 6 weeks) |
| 64 | -- |
| 65 | -- |
| 66 | -- |
| 67 | -- |
| 68 | -- |
| 69 | -- |
| 70 | -- |
| 71 | PDE5i (daily for 12 weeks or on demand) |
| 72 | PDE5i (daily for 4 weeks) |
| 73 | -- |
| 74 | -- |
| 75 | --- |
| 76 | -- |
| 77 | -- |
| 78 | -- |
| 79 | -- |
| 80 | -- |
| 81 | -- |
| 82 | -- |
| 83 | -- |
| 84 | -- |
| 85 | PDE5i (daily for 4 weeks) |
| 86 | -- |
| 87 | -- |

| **Ref** | **V32** | **V33** | **V34^[[26]](#footnote-26)^** | **V34^[[27]](#footnote-27)^** | **V34^[[28]](#footnote-28)^** | **V34^[[29]](#footnote-29)^** | **V35^26^** | **V35^27^** | **V35^28^** | **V35^[[30]](#footnote-30)^** | **V36** |
| --- | --- | --- | --- | --- | --- | --- | --- | --- | --- | --- | --- |
| 1 | 42 | 0 | 57.5 |  | 6.9 |  | 51.2 |  | 8.4 |  | ESWT (6 sessions; W1-W3 (2)) + 5 mg of tadalafil daily |
| 2 | 17 | 0 | 58.4 |  | 7 |  | 65 |  | 48 |  | Sham ESWT + PDE5i (daily from W1-W4) |
| 5 | 34 | 0 | 35.3 |  |  | 25-50 | 23.4 |  |  | 6-84 | PDE5i + L-arginine |
| 7 | 62 | 32 | 62.5 |  | 7.6 |  | -- |  | -- |  | Standard care |
| 9 | 59 | 20 |  | 66 |  |  | -- |  | -- |  | PDE5i (daily for 6 months post-OP) |
| 10 | 16 | 1 |  | 64.4 |  |  | n.s. |  |  |  | Sham ESWT |
| 11 | 13 | **^[[31]](#footnote-31)^** |  | 61 |  | 49-69 | -- |  | -- |  | PDE5i (2x per week for >4 weeks) |
| 12 | 484 | 0 | 52.5 |  | 11.6 |  | n.s. |  |  |  | ESWT (Renova/Direx; W1-W4 (1); 3600 shots, 0,09 mJ/mm^2^, 5 Hz) |
| 15 | 44 | 14 | 31.2 |  | 5.2 |  | 12 |  |  | 11-24 | PDE5i on demand |
| 19 | 50 | 8 | 49.6 |  | 14 |  |  |  |  | >3 | ESWT (6 sessions; W1-W6 (1) |
| 21 | 35 | 2 |  | 61 |  |  |  | 48 |  |  | Sham ESWT |
| 22 | 20 | 4 | 60 |  | 5 |  | 7 |  |  | 3-18 | Sham ESWT |
| 23 | 25 | 0 |  | 58 |  |  |  | 56 |  |  | ESWT (same protocol) + placebo PDE5i (daily from W1-W4) |
| 25 | 24 | 0 |  | 55 |  |  | n.s. |  |  |  | Sham ESWT |
| 26 | 40 | 1 | 48.5 |  | 11.2 |  | 4.7 |  | 4.6 |  | Sham ESWT |
| 28 | 35 | 2 | 46.7 |  | 4.9 |  | 43.9 |  | 27.2 |  | ESWT (same protocol) |
| 29 | 26 | 1 | 47.1 |  |  | 32-63 | 91 |  |  | 24-180 | PDE5i on demand |
| 34 | 93 | 0 | 51.2 |  | 11.4 |  | 48 |  | 36 |  | ESWT (5x; W1-W5 (1)) |
| 35 | 32 | 0 | 58.4 |  |  | 49-70 | -- |  | -- |  | PDE5i (daily) |
| 36 | 18 | 0 | 64.3 |  | 4.8 |  | 14.3 |  | 16.4 |  | Sham ESWT |
| 38 | 40 | 8 | 30.7 |  | 4.2 |  | 15 |  |  | 6-24 | PDE5i on demand |
| 39 | 22 | 22 | 39.9 |  | 11.6 |  | 37.2 |  | 46.3 |  | Sham ESWT |
| 41 | 21 | 0 | 47.5 |  | 5.7 |  |  |  |  | >6 | Kegel exercises three times daily for 6 weeks |
| 43 | 40 | 4 |  | 60 |  | 53-64 |  | 54 |  | 36-72 | Sham ESWT |
| 44 | 46 | 5 | 64.4 |  | 5.3 |  | -- |  | -- |  | PDE5i (daily for 8 weeks post-OP) |
| 45 | 20 | 0 | 44.3 |  | n.s. |  | n.s. |  |  |  | fESWT (W1-W3 and W7-W9 (1)) |
| 47 | 24 | 2 | 57.7 |  | 9.3 |  |  |  |  | >6 | ESWT (W1-W4 (3), EFD = 0.05 mJ/mm^2^, 8 Hz) |
| 48 | 20 | 0 | 59.2 |  | 8.7 |  | n.s. |  |  |  | Only ESWT (same protocol; no TMS) |
| 50 | 11 | **^[[32]](#footnote-32)^** | 62.9 |  | 1.8 |  | -- |  | -- |  | ESWT (12x; W1-W3 and W7-W9 (2) |
| 51 | 49 | 6 | 65.1 |  | 7.9 |  |  |  |  | >6 | Sham ESWT |
| 52 | 42 | 2 | 50.5 |  |  | 30-78 |  |  |  | >6 | ESWT (W1-W2 (3), 600 ESWs each, not further specified) |
| 53 | 78 | 0 | 58.2 |  | 3.2 |  | n.s. |  |  |  | PDE5i (daily for 12 weeks) |
| 55 | 24 | 0 |  | 61 |  | 54-69 |  | 68 |  | 12-96 | Radial ESWT (Zimmer enPulse, W1-W6 (1), 10.000 rESWs, 15 Hz, 90 mJ) |
| 56 | 20 | 0 | 51 |  |  | 45-60 | n.s. |  |  |  | Chorionic gonadotropin + ICA (no ESWT) |
| 59 | 30 | 0 | 54.7 |  | 9.2 |  | 45 |  |  | 6-204 | Sham ESWT |
| 62 | 10 | 0 | 52.2 |  |  | 46-61 | 32.8 |  | 23.7 |  | Sham ESWT |
| 64 | 63 | 20 | 63.3 |  | 9.5 |  |  |  |  | >6 | Sham ESWT |
| 65 | 22 | 4 | 55.6 |  | 9 |  |  |  |  | >3 | ESWT (W1-W6 (2) and W25-W30 (1)) |
| 67 | 53 | 10 | 51.2 |  | 6.3 |  | -- |  | -- |  | Sham ESWT |
| 69 | 63 | 3 | 63.3 |  | 9.5 |  |  |  |  | >6 | Sham ESWT |
| 70 | 16 | 0 | 55.1 |  |  | 38-72 | 66 |  |  | 12-180 | Sham ESWT |
| 75 | 18 | 0 |  | 64 |  | 29-81 | 72 |  |  | 8-180 | Sham ESWT |
| 78 | 54 | 14 |  | 60 |  | 37-79 |  | 64 |  | 12-240 | Sham ESWT |
| 82 | 40 | 23 | n.s. |  |  |  | n.s. |  |  |  | Sham ESWT |
| 83 | 34 | 6 | 63.3 |  | 6.4 |  | 89 |  | 52 |  | Sham ESWT |
| 85 | 50 | 0 | 54 |  |  | 29-71 | 9 |  |  | 7-12 | ESWT (same protocol) |
| 86 | 21 | 1 |  | 57 |  | 35-77 |  | 60 |  | 6-240 | Sham ESWT |

| **Ref** | **V37** | **V38** | **V39^26^** | **V39^27^** | **V39^28^** | **V39^29^** | **V40^26^** | **V40^27^** | **V40^28^** | **V40^30^** | **V41** |
| --- | --- | --- | --- | --- | --- | --- | --- | --- | --- | --- | --- |
| 1 | 27 | 0 | 54.1 |  | 6.5 |  |  |  |  | >6 | ESWT (6 sessions; W1-W3 (2)) |
| 5 | 34 | 0 | 35.3 |  |  | 25-50 | 23.4 |  |  | 6-84 | PDE5i |
| 11 | 139 | **^31^** |  | 66 |  | 46-75 | -- | -- |  |  | No treatment |
| 28 | 35 | 1 | 47.9 |  | 5.7 |  | 45.5 | 22 |  |  | Vacuum erectile device (same protocol) |
| 47 | 24 | 1 | 56.5 |  | 7.6 |  |  |  |  | >6 | ESWT (W1-W6 (2), EFD = 0,1 mJ/mm2, 5 Hz) |
| 50 | 178 | **^32^** | 66.6 |  | 0.45 |  | -- | -- |  |  | None |

| **Ref** | **V42** | **V43** | **V44^26^** | **V44^27^** | **V44^28^** | **V44^29^** | **V45^26^** | **V45^27^** | **V45^28^** | **V45^30^** | **V46** |
| --- | --- | --- | --- | --- | --- | --- | --- | --- | --- | --- | --- |
| 47 | 25 | 4 | 57.6 | 6.9 |  |  |  |  |  | >6 | ESWT (W1-W4 (3), EFD = 0,1 mJ/mm^2^, 5 Hz) |

| **Ref** | **V47** | **V48** | **V49** | **V50** | **V51** | **V52^[[33]](#footnote-33)^** | **V52^[[34]](#footnote-34)^** | **V52^[[35]](#footnote-35)^** | **V52^[[36]](#footnote-36)^** | **V53** | V54 | V55 |
| --- | --- | --- | --- | --- | --- | --- | --- | --- | --- | --- | --- | --- |
| 1 | Yes | No | n.a. | n.a. | No |  |  |  |  | No | Yes |  |
| 2 | Yes | No | n.a. | n.a. | No | 7.7 | 1.3 | 12.9 | 2.1 | Yes | Yes | A |
| 3 | Yes | No | n.a. | n.a. | No |  |  |  |  | n.a. | Yes |  |
| 4 | Yes | No | n.a. | n.a. | No |  |  |  |  | n.a. | Yes |  |
| 5 | Yes | No | n.a. | n.a. | No | 14.7 | 1.5 | 25.8 | 3.6 | Yes | Yes | B |
| 6 | Yes | No | n.a. | n.a. | No |  |  |  |  | n.a. | Yes |  |
| 7 | No | Yes | No | n.a. | No |  |  |  |  | n.a. | n.a. |  |
| 8 | Yes | No | n.a. | n.a. | No |  |  |  |  | n.a. | Yes |  |
| 9 | No | Yes | No | n.a. | No |  |  |  |  | n.a. | n.a. |  |
| 10 | No | Yes | No | n.a. | No |  |  |  |  | Yes | Yes | A |
| 11 | No | Yes | No | n.a. | No |  |  |  |  | n.a. | n.a. |  |
| 12 | Yes | No | n.a. | n.a. | No |  |  |  |  | No | Yes |  |
| 13 | Yes | No | n.a. | n.a. | No |  |  |  |  | n.a. | Yes |  |
| 14 | No | Yes | No | n.a. | No | 14.3 | 5.2 | 20.3 | 8.4 | n.a. | Yes |  |
| 15 | No | Yes | No | n.a. | No | 8.6 | 3.6 | 16.3 | 5.5 | No | Yes | C |
| 16 | Yes | No | n.a. | n.a. | No |  |  |  |  | n.a. | Yes |  |
| 17 | Yes | No | n.a. | n.a. | No |  |  |  |  | n.a. | Yes |  |
| 18 | Yes | No | n.a. | n.a. | No |  |  |  |  | n.a. | No | B |
| 19 | No | Yes | No | n.a. | No | 16.5 | 4.1 | 21.5 | 4.5 | No | Yes |  |
| 20 | Yes | No | n.a. | n.a. | No | 15.17 | 3.75 | 21.61 | 3.6 | n.a. | Yes |  |
| 21 | No | Yes | No | n.a. | No | 14 | 1.7 | 20 | 2.4 | Yes | Yes | B |
| 22 | No | Yes | No | n.a. | No |  |  |  |  | Yes | n.a. | C |
| 23 | Yes | No | n.a. | n.a. | No | 21.2 | 2.5 | 25.3 | 2.3 | No | Yes |  |
| 24 | Yes | No | n.a. | n.a. | No |  |  |  |  | n.a. | Yes |  |
| 25 | Yes | No | n.a. | n.a. | No |  |  |  |  | Yes | Yes | A |
| 26 | Yes | Yes | **Yes** | BOCF | No | 16.2 | 0.6 | 17 | 1.2 | Yes | No | D |
| 27 | Yes | No | n.a. | n.a. | No |  |  |  |  | n.a. | Yes |  |
| 28 | No | Yes | No | n.a. | No | 13.3 | 1.6 | 18.9 | -- | No | Yes | A |
| 29 | No | Yes | No | n.a. | No |  |  |  |  | No | Yes | C |
| 30 | Yes | No | n.a. | n.a. | No |  |  |  |  | n.a. | Yes |  |
| 31 | No | Yes | No | n.a. | No |  |  |  |  | n.a. | Yes |  |
| 32 | Yes | No | n.a. | n.a. | No |  |  |  |  | n.a. | Yes |  |
| 33 | Yes | No | n.a. | n.a. | No |  |  |  |  | n.a. | No |  |
| 34 | Yes | No | n.a. | n.a. | No | 14.3 | 4.4 | 23.8 | 4.4 | No | Yes |  |
| 35 | Yes | No | n.a. | n.a. | No |  |  |  |  | Yes | n.a. | A |
| 36 | Yes | No | n.a. | n.a. | No |  |  |  |  | Yes | n.a. | A |
| 37 | No | Yes | No | n.a. | No |  |  |  |  | n.a. | Yes |  |
| 38 | No | Yes | No | n.a. | No |  |  |  |  | No | Yes | C |
| 39 | Yes | Yes | **Yes** | BOCF | No | 20.32 | 2.32 | 23.1 | 2.82 | No | Yes | A |
| 40 | No | Yes | No | n.a. | No | 13.5 | 4.6 | 22.1 | 5.3 | n.a. | Yes |  |
| 41 | Yes | No | n.a. | n.a. | No | 12.8 | 3.2 | 17.5 | 2.7 | Yes | Yes | A |
| 42 | Yes | No | n.a. | n.a. | No |  |  |  |  | n.a. | Yes |  |
| 43 | No | Yes | No | n.a. | No | 12 | 8-17**^[[37]](#footnote-37)^** | 15 | 7-22**^37^** | No | Yes | A |
| 44 | No | Yes | No | n.a. | No |  |  |  |  | n.a. | n.a. |  |
| 45 | Yes | No | n.a. | n.a. | No |  |  |  |  | No | Yes |  |
| 46 | Yes | No | n.a. | n.a. | No |  |  |  |  | n.a. | Yes |  |
| 47 | No | Yes | No | n.a. | No | 18.3 | 4.2 | 23.6 | 4 | No | Yes |  |
| 48 | Yes | No | No | n.a. | No |  |  |  |  | No | Yes |  |
| 49 | No | Yes | No | n.a. | No |  |  |  |  | n.a. | Yes |  |
| 50 | No | Yes | No | n.a. | No |  |  |  |  | n.a. | n.a. |  |
| 51 | No | Yes | No | n.a. | No | 16.6 | 3 | 21.7 | 3 | Yes | Yes | A |
| 52 | No | Yes | No | n.a. | No | 18.1 | 17.1-19.1**^[[38]](#footnote-38)^** | 21 | 19.4-22.6**^38^** | No | Yes |  |
| 53 | Yes | No | n.a. | n.a. | No |  |  |  |  | Yes | Yes | B |
| 54 | Yes | No | n.a. | n.a. | No |  |  |  |  | n.a. | Yes |  |
| 55 | Yes | No | n.a. | n.a. | No |  |  |  |  | No | Yes |  |
| 56 | Yes | No | n.a. | n.a. | No |  |  |  |  | No | Yes |  |
| 57 | Yes | No | n.a. | n.a. | No |  |  |  |  | n.a. | Yes |  |
| 58 | No | Yes | No | n.a. | No |  |  |  |  | n.a. | Yes |  |
| 59 | Yes | No | n.a. | n.a. | No |  |  |  |  | Yes | Yes | A |
| 60 | Yes | No | n.a. | n.a. | No |  |  |  |  | n.a. | Yes |  |
| 61 | Yes | No | n.a. | n.a. | No |  |  |  |  | n.a. | Yes |  |
| 62 | Yes | No | n.a. | n.a. | No |  |  |  |  | Yes | Yes | A |
| 63 | Yes | No | n.a. | n.a. | No |  |  |  |  | n.a. | Yes |  |
| 64 | No**^[[39]](#footnote-39)^** | Yes | No | n.a. | No | 11.2 | 6.6 | 16 | 9.8 | No | Yes |  |
| 65 | No | Yes | No | n.a. | No | 16.2 | 3.8 | 19.3 | 4 | No | Yes |  |
| 66 | Yes | No | n.a. | n.a. | No |  |  |  |  | n.a. | Yes |  |
| 67 | No | Yes | No | n.a. | No |  |  |  |  | n.a. | n.a. |  |
| 68 | No | Yes | No | n.a. | No |  |  |  |  | n.a. | Yes |  |
| 69 | No | Yes | No | n.a. | No | 10.9 | 9.1-12.7**^38^** | 13.1 | 9.3-13.4**^38^** | Yes | No | D |
| 70 | Yes | No | n.a. | n.a. | No | 13.8 | 3.6 | 19 | 3.3 | Yes | Yes | A |
| 71 | Yes | No | n.a. | n.a. | No |  |  |  |  | n.a. | Yes |  |
| 72 | No | Yes | No | n.a. | No |  |  |  |  | n.a. | Yes |  |
| 73 | No | Yes | No | n.a. | Yes**^[[40]](#footnote-40)^** |  |  |  |  | n.a. | n.a. |  |
| 74 | Yes | No | n.a. | n.a. | No |  |  |  |  | n.a. | Yes |  |
| 75 | No | Yes | No | n.a. | No | 7 | 6-10**^37^** | 13 | 9-18**^37^** | No | Yes | A |
| 76 | Yes | No | n.a. | n.a. | No | 14.9 | -- | 19.9 | -- | n.a. | Yes |  |
| 77 | Yes | No | n.a. | n.a. | No |  |  |  |  | n.a. | No |  |
| 78 | No | Yes | No | n.a. | No |  |  |  |  | No | No |  |
| 79 | Yes | No | n.a. | n.a. | No | 15 | -- | 20 | -- | n.a. | Yes |  |
| 80 | Yes | No | n.a. | n.a. | No | 14.8 | 4.8 | 22.3 | -- | n.a. | Yes |  |
| 81 | Yes | No | n.a. | n.a. | No | 16.5 | 6.4 | 21 | 6.4 | n.a. | Yes |  |
| 82 | No | Yes | No | n.a. | No | 9.5 | 2 | 22 | 3 | Yes | Yes | A |
| 83 | No | Yes | No | n.a. | No | 10.2 | 3.8 | 17.8 | 4.8 | Yes | Yes | C |
| 84 | No | Yes | No | n.a. | No | 8.8 | -- | 12.3 | -- | n.a. | Yes |  |
| 85 | Yes | No | n.a. | n.a. | No | 11.6 |  | 21.5 |  | No | Yes | B |
| 86 | No | Yes | No | n.a. | No | 11.5 | 0.86 | 18.2 | 0.8 | Yes | Yes | B |
| 87 | Yes | No | n.a. | n.a. | No | 13.5 | 4.1 | 20.9 | 5.8 | n.a. | Yes |  |

**REFERENCES**

1. İbis MA, Ozkaya F, Tokatli Z, Akpinar C, Yaman O. Efficacy of low-intensity shockwave therapy with different tadalafil regimens in patients with PDE5 inhibitor-resistant erectile dysfunction: a retrospective cohort study. *Int Urol Nephrol* 2024;56:407-413. https://doi.10.1007/s11255-023-03769-w.

2. Kalyvianakis D, Mykoniatis I, Pyrgidis N, Kapoteli P, Zilotis F, Hatzichristou D. The effect of combination treatment with low-intensity shockwave therapy and daily tadalafil on severe erectile dysfunction: a double-blind, randomized, sham-controlled clinical trial. *J Sex Med* 2024;21:533-538. https://doi.10.1093/jsxmed/qdae038.

3. Kaynak Y, Gruenwald I. Long-term effects of combination treatment comprising low-intensity extracorporeal shockwave therapy and tadalafil for patients with erectile dysfunction: a retrospective study. *Int J Impot Res* 2024;36:601-606. https://doi.10.1038/s41443-023-00757-7.

4. Rubino M, Ricapito A, Finati M et al. Impact of low-intensity extracorporeal shockwave therapy on vascular parameters and sexual function in patients with arteriogenic erectile dysfunction. *Asian J Androl* 2024;26:344-348. https://doi.10.4103/aja202384.

5. Trishch VI, Mysak AI, Trishch AI, Mandzii AP. Assessment of the treatment effectiveness of men with mild and medium degree of erectile dysfunction. *Pol Merkur Lekarski* 2024;52:79-86. https://doi.10.36740/Merkur202401113.

6. Bayraktar N. Non-invasive alternative for phosphodiesterase inhibitor-refractory erectile dysfunction: Real-life experience with low-intensity extracorporeal shockwave therapy. *Medicine* 2023;102:e35939. https://doi.10.1097/MD.0000000000035939.

7. Bryk DJ, Murthy PB, Ericson KJ, Shoskes DA. Radial wave therapy does not improve early recovery of erectile function after nerve-sparing radical prostatectomy: a prospective trial. *Transl Androl Urol* 2023;12:209-216. https://doi.10.21037/tau-22-310.

8. Islam R, Rahaman KS, Hawlader MDH. Efficacy of low-intensity extra corporal shockwave therapy (LI-ESWT) in patients with erectile dysfunction. *J Family Reprod Health* 2023;17:93-99. https://doi.10.18502/jfrh.v17i2.12872.

9. Jang SW, Lee EH, Chun SY et al. Comparison of the efficacy of the early LI-SWT plus daily tadalafil with daily tadalafil only as penile rehabilitation for postprostatectomy erectile dysfunction. *Int J Impot Res* 2023;35:447-453. https://doi.10.1038/s41443-022-00560-w.

10. Kennady EH, Bryk DJ, Ali MM et al. Low-intensity shockwave therapy improves baseline erectile function: a randomized sham-controlled crossover trial. *Sex Med* 2023;11:qfad053. https://doi.10.1093/sexmed/qfad053.

11. Kohada Y, Babasaki T, Goto K et al. Long-term efficacy of penile rehabilitation with low-intensity extracorporeal shock wave therapy for sexual and erectile function recovery following robotic-assisted radical prostatectomy: a single-cohort pilot study. *Sex Med* 2023;11:qfad023. https://doi.10.1093/sexmed/qfad023.

12. Kurosawa M, Tsujimura A, Morino J et al. Efficacy and patient satisfaction of low-intensity shockwave treatment for erectile dysfunction in a retrospective real-world study in Japan. *Int J Urol* 2023;30:375-380. https://doi.10.1111/iju.15135.

13. Ma MF, He JH, Zhao XD, et al. [Low-intensity extracorporeal shockwave therapy improves symptoms of erectile dysfunction: A preliminary study]. *Zhonghua Nan Ke Xue* 2023;29:239-243. Chinese.

14. Vena W, Vaccalluzzo L, LA Vignera S, et al. Low-intensity shockwave treatment (LISWT) improves penile rigidity in eugonadal subjects with erectile dysfunction: a pilot study. *Minerva Endocrinol* 2023;48:4-11. https://doi.10.23736/S2724-6507.21.03686-1.

15. Wang D, Wang SJ, Li YJ, et al. The treatment satisfaction in patients and their partners treated with low-intensity extracorporeal shock wave therapy and sildenafil: a prospective non-randomized controlled study. *Patient Prefer Adherence* 2023;17:583-589. https://doi.10.2147/PPA.S399776.

16. Cocci A, Fassio G, Migliorini F et al. Low-intensity extracorporeal shock wave therapy (Li-ESWT) for priapism-induced erectile dysfunction in young patients: the first case series*. Int J Impot Res* 2022;34:277-279. https://doi.10.1038/s41443-021-00429-4.

17. Daneshwar D, Nordin A. Low intensity extracorporeal shockwave therapy for chronic pelvic pain syndrome patients with erectile dysfunction. *Medicine* 2022;101:e28546. https://doi.10.1097/MD.0000000000028546.

18. Ergün M, Akyüz O. Is Li-ESWT effective in diabetic patients with severe erectile dysfunction? *Asian J Androl* 2022;24:521-524. https://doi.10.4103/aja2021105.

19. Gallo L, Pecoraro S, Sarnacchiaro P. Adjuvant daily therapy with L-arginine 2,500 mg and tadalafil 5 mg increases efficacy and duration of benefits of low-intensity extracorporeal shock wave therapy for erectile dysfunction: A prospective, randomized, single-blinded study with 1-year follow-up. *Investig Clin Urol* 2022;63:83-91. https://doi.10.4111/icu.20210317.

20. Geyik S. A single-centre result of two courses of low-intensity shockwave therapy (Li-SWT) in erectile dysfunction. *Andrologia* 2022;54:e14324. https://doi.10.1111/and.14324.

21. Kalyvianakis D, Mykoniatis I, Pyrgidis N et al. The effect of low-intensity shock wave therapy on moderate erectile dysfunction: a double-blind, randomized, sham-controlled clinical trial. *J Urol* 2022;208:388-395. https://doi.10.1097/JU.0000000000002684.

22. Motil I, Macik D, Sramkova K, Jarkovsky J, Sramkova T. Linear low-intensity extracorporeal shockwave therapy as a method for penile rehabilitation in erectile dysfunction patients after radical prostatectomy: a randomized, single-blinded, sham-controlled clinical trial. *Urol Int* 2022;106:1050-1055. https://doi.10.1159/000525973.

23. Mykoniatis I, Pyrgidis N, Zilotis F et al. The effect of combination treatment with low-intensity shockwave therapy and tadalafil on mild and mild-to-moderate erectile dysfunction: a double-blind, randomized, placebo-controlled clinical trial. *J Sex Med* 2022;19:106-115. https://doi.10.1016/j.jsxm.2021.10.007.

24. Oginski N, Apel H, Richterstetter M et al. Analysis of the impact of clinical factors on low-intensity extracorporeal shockwave therapy for erectile dysfunction. *Urol Int* 2022;106:1041-1049. https://doi.10.1159/000520705.

25. Ong WLK, Lechmiannandan S, Lim YL, Manoharan D, Lee SB. Early outcomes of short-course low intensity shockwave therapy (LiSWT) for erectile dysfunction: A prospective, randomized, double-blinded, sham-controlled study in Malaysia. *Andrologia* 2022;54:e14518. https://doi.10.1111/and.14518.

26. Sandoval-Salinas C, Saffon JP, Martínez JM, Corredor HA, Gallego A. Are radial pressure waves effective for the treatment of moderate or mild to moderate erectile dysfunction? A randomized sham therapy controlled clinical trial. *J Sex Med* 2022;19:738-744. https://doi.10.1016/j.jsxm.2022.02.010.

27. Scroppo FI, Pezzoni F, Gaeta F et al. LI-ESWT improves hemodynamic parameters thus suggesting neoangiogenesis in patients with vascular erectile dysfunction*. Int J Impot Res* 2022;34:237-242. https://doi.10.1038/s41443-021-00411-0.

28. Tao R, Chen J, Wang D, et al. The efficacy of Li-ESWT combined with ved in diabetic ed patients unresponsive to pde5is: a single-center, randomized clinical trial. *Front Endocrinol* 2022;13:937958. https://doi.10.3389/fendo.2022.937958.

29. Zanaty F, Badawy A, Kotb H, Elsarfy F, Salman B. Efficacy and safety of low-intensity extracorporeal shock wave therapy versus on-demand tadalafil for erectile dysfunction. *Arab J Urol* 2022;20:189-194. https://doi.10.1080/2090598X.2022.2090134.

30. Adeldaeim HM, Abouyoussif T, Gebaly OE et al. Prognostic indicators for successful low-intensity extracorporeal shock wave therapy treatment of erectile dysfunction. *Urology* 2021;149:133-139. https://doi.10.1016/j.urology.2020.12.019.

31. Akande TO, Akinwunmi OM, Adebayo SA, Akinyinka AO, Shittu OB. Efficacy and safety of low-intensity extracorporeal shockwave therapy for treatment of vascular erectile dysfunction in nigerian men: report of a study in ibadan, south-west nigeria. *Ann Ib Postgrad Med* 2021;19:8-14.

32. Caretta N, De Rocco Ponce M, Minicuci N et al. Efficacy of penile low-intensity shockwave treatment for erectile dysfunction: correlation with the severity of cavernous artery disease. *Asian J Androl* 2021;23:462-467. https://doi.10.4103/aja.aja_15_21.

33. Chung E, Cartmill R. Evaluation of long-term clinical outcomes and patient satisfaction rate following low intensity shock wave therapy in men with erectile dysfunction: a minimum 5-year follow-up on a prospective open-label single-arm clinical study. *Sex Med* 2021;9:100384. https://doi.10.1016/j.esxm.2021.100384.

34. Geyik S. Comparison of the efficacy of low-intensity shock wave therapy and its combination with platelet-rich plasma in patients with erectile dysfunction. *Andrologia* 2021;53:e14197. https://doi.10.1111/and.14197.

35. Karakose A, Yitgin Y. Penile rehabilitation with low-intensity extracorporeal shock wave therapy in patients after prostate cancer surgery. Early physiological changes and postoperative follow-up outcomes. *Int J Clin Pract* 2021;75:e14804. https://doi.10.1111/ijcp.14804.

36. Ladegaard PBJ, Mortensen J, Skov-Jeppesen SM, Lund L. Erectile dysfunction a prospective randomized placebo-controlled study evaluating the effect of low-intensity extracorporeal shockwave therapy (LI-ESWT) in men with erectile dysfunction following radical prostatectomy. *Sex Med* 2021;9:100338. https://doi.10.1016/j.esxm.2021.100338.

37. Lau W, Shum CF, Lua HCA, Teo CPC. Low-intensity shockwave therapy in the management of erectile dysfunction in Singapore. *Ann Acad Med Singap* 2021;50:652-654. https://doi.10.47102/annals-acadmedsg.202151.

38. Lei Q, Wang D, Liu C, Ji Z, Yan S. Comparison of the efficacy and safety of low-intensity extracorporeal shock wave therapy versus on-demand sildenafil for erectile dysfunction. *Transl Androl Urol* 2021;10:860-868. https://doi.10.21037/tau-20-1069.

39. Ortac M, Özmez A, Cilesiz NC, Demirelli E, Kadıoğlu A. The impact of extracorporeal shock wave therapy for the treatment of young patients with vasculogenic mild erectile dysfunction: A prospective randomized single-blind, sham controlled study. *Andrology* 2021;9:1571-1578. https://doi.10.1111/andr.13007.

40. Palmieri A, Arcaniolo D, Palumbo F et al. Low intensity shockwave therapy in combination with phosphodiesterase-5 inhibitors is an effective and safe treatment option in patients with vasculogenic ED who are PDE5i non-responders: a multicenter single-arm clinical trial. *Int J Impot Res* 2021;33:634-640. https://doi.10.1038/s41443-020-0332-7.

41. Shendy WS, Elsoghier OM, El Semary MM et al. Effect of low-intensity extracorporeal shock wave therapy on diabetic erectile dysfunction: Randomised control trial. *Andrologia* 2021;53:e13997. https://doi.10.1111/and.13997.

42. Tzou KY, Hu SW, Bamodu OA, Wang YH, Wu WL, Wu CC. Efficacy of penile low-intensity shockwave therapy and determinants of treatment response in taiwanese patients with erectile dysfunction. *Biomedicines* 2021;9:1670. https://doi.10.3390/biomedicines9111670.

43. Vinay J, Moreno D, Rajmil O, Ruiz-Castañe E, Sanchez-Curbelo J. Penile low intensity shock wave treatment for PDE5I refractory erectile dysfunction: a randomized double-blind sham-controlled clinical trial. *World J Urol* 2021;39:2217-2222. https://doi.10.1007/s00345-020-03373-y.

44. Baccaglini W, Pazeto CL, Corrêa Barros EA, et al. The role of the low-intensity extracorporeal shockwave therapy on penile rehabilitation after radical prostatectomy: a randomized clinical trial. *J Sex Med* 2020;17:688-694. https://doi.10.1016/j.jsxm.2019.12.024.

45. Eryilmaz R, Kaplan Ş, Aslan R, Demir M, Taken K. Comparison of focused and unfocused ESWT in treatment of erectile dysfunction. *Aging Male* 2020;23:206-209. https://doi.10.1080/13685538.2019.1610377.

46. Huang YP, Liu W, Liu YD, Zhang M, Xu SR, Lu MJ. Effect of low-intensity extracorporeal shockwave therapy on nocturnal penile tumescence and rigidity and penile haemodynamics. *Andrologia* 2020;52:e13745. https://doi.10.1111/and.13745.

47. Kalyvianakis D, Mykoniatis I, Memmos E, Kapoteli P, Memmos D, Hatzichristou D. Low-intensity shockwave therapy (LiST) for erectile dysfunction: a randomized clinical trial assessing the impact of energy flux density (EFD) and frequency of sessions. *Int J Impot Res* 2020;32:329-337. https://doi.10.1038/s41443-019-0185-0.

48. Konchugova TV, Kulchitskaya DB, Kiyatkin VA, Gushchina NV. [Transcerebral magnetic and shock wave therapy in correction of erectile dysfunction]. *Vopr Kurortol Fizioter Lech Fiz Kult* 2020;97:60-67. Russian. https://doi.10.17116/kurort20209703160.

49. Lurz K, Dreher P, Levy J et al. Low-intensity shockwave therapy in the treatment of erectile dysfunction. *Cureus* 2020;12:e11286. https://doi.10.7759/cureus.11286.

50. Inoue S, Hayashi T, Teishima J, Matsubara A. Effect of penile rehabilitation with low intensity extracorporeal shock wave therapy on erectile function recovery following robot-assisted laparoscopic prostatectomy. *Transl Androl Urol* 2020;9:1559-1565. https://doi.10.21037/tau-19-888.

51. Kim KS, Jeong HC, Choi SW et al. Electromagnetic low-intensity extracorporeal shock wave therapy in patients with erectile dysfunction: a sham-controlled, double-blind, randomized prospective study. *World J Mens Health* 2020;38:236-242. https://doi.10.5534/wjmh.190130.

52. Patel P, Katz J, Lokeshwar SD et al. Phase II randomized, clinical trial evaluating 2 schedules of low-intensity shockwave therapy for the treatment of erectile dysfunction. *Sex Med* 2020;8:214-222. https://doi.10.1016/j.esxm.2020.01.010.

53. Verze P, Capece M, Creta M et al. Efficacy and safety of low-intensity shockwave therapy plus tadalafil 5 mg once daily in men with type 2 diabetes mellitus and erectile dysfunction: a matched-pair comparison study. *Asian J Androl* 2020;22:379-382. https://doi.10.4103/aja.aja_121_19.

54. Wang J, Luo L, Zhao S, Liu Y, Zhu Z, Zhao Z. Low intensity extracorporeal shockwave therapy shifts PDE5i nonresponders to responders. *Int Braz J Urol* 2020;46:934-942. https://doi.10.1590/S1677-5538.IBJU.2019.0374.

55. Wu SS, Ericson KJ, Shoskes DA. Retrospective comparison of focused shockwave therapy and radial wave therapy for men with erectile dysfunction. *Transl Androl Urol* 2020;9:2122-2128. https://doi.10.21037/tau-20-911.

56. Zasieda Y. Combined treatment with focused low-intensity shock-wave therapy and androgen-stimulation therapy in men with corporal veno-occlusive erectile dysfunction on the background of hypogonadotropic hypogonadism. *Georgian Med News* 2020;:49-53.

57. Costa P, Dias J, Gouveia R, et al. Low intensity extracorporeal shockwave therapy on erectile dysfunction—first results from a prospective study. *AME Med J* 2019;4:32. https://doi.10.21037/amj.2019.06.03.

58. Musa ZS, El-Assmy A, Shokry AM, Shokeir AA, Zween T, Al-Kenawy MR. Long-term effectiveness and predictors of success of low-intensity shockwave therapy in phosphodiesterase type 5 inhibitors non-responders. *Arab J Urol* 2019;18:54-58. https://doi.10.1080/2090598X.2019.1688072.

59. Sramkova T, Motil I, Jarkovsky J, Sramkova K. Erectile dysfunction treatment using focused linear low-intensity extracorporeal shockwaves: single-blind, sham-controlled, randomized clinical trial. *Urol Int* 2020;104:417-424. https://doi.10.1159/000504788.

60. Vita R, Benvenga S, Giammusso B, La Vignera S. Determinants of early response to low-intensity extracorporeal shockwaves for the treatment of vasculogenic erectile dysfunction: an open-label, prospective study. *J Clin Med* 2019;8:1017. https://doi.10.3390/jcm8071017.

61. Wang CJ, Lu YM, Li CC, Wu WJ, Chien TM. Low-intensity shock wave therapy ameliorates erectile dysfunction in men with pelvic fractures associated with urethral injury. *Int J Impot Res* 2019;31:218-222. https://doi.10.1038/s41443-018-0094-7.

62. Yamaçake KGR, Carneiro F, Cury J, et al. Low-intensity shockwave therapy for erectile dysfunction in kidney transplant recipients. A prospective, randomized, double blinded, sham-controlled study with evaluation by penile Doppler ultrasonography. *Int J Impot Res* 2019;31:195-203. https://doi.10.1038/s41443-018-0062-2.

63. De Oliveira PS, De Oliveira TR, Nunes Á, Martins F, Lopes T. Low-intensity shock wave therapy for erectile dysfunction and the influence of disease duration. *Arch Ital Urol Androl* 2019;90:276-282. https://doi.10.4081/aiua.2018.4.276.

64. Fojecki GL, Tiessen S, Osther PJS. Effect of linear low-intensity extracorporeal shockwave therapy for erectile dysfunction-12-month follow-up of a randomized, double-blinded, sham-controlled study. *Sex Med* 2018;6:1-7. https://doi.10.1016/j.esxm.2017.09.002.

65. Kalyvianakis D, Memmos E, Mykoniatis I, Kapoteli P, Memmos D, Hatzichristou D. Low-intensity shockwave therapy for erectile dysfunction: a randomized clinical trial comparing 2 treatment protocols and the impact of repeating treatment. *J Sex Med* 2018;15:334-345. https://doi.10.1016/j.jsxm.2018.01.003.

66. Kitrey ND, Vardi Y, Appel B, et al. Low intensity shock wave treatment for erectile dysfunction – how long does the effect last? *J Urol* 2018l;200:167-170. https://doi.10.1016/j.juro.2018.02.070.

67. Zewin TS, El-Assmy A, Harraz AM, et al. Efficacy and safety of low-intensity shock wave therapy in penile rehabilitation post nerve-sparing radical cystoprostatectomy: a randomized controlled trial. *Int Urol Nephrol* 2018;50:2007-2014. https://doi.10.1007/s11255-018-1987-6.

68. Ayala HAC, Cuartas JPS, Cleves DC. Impact on the quality of erections after completing a low-intensity extracorporeal shock wave treatment cycle on a group of 710 patients. *Adv Urol* 2017;2017:1843687. https://doi.10.1155/2017/1843687.

69. Fojecki GL, Tiessen S, Osther PJ. Effect of low-energy linear shockwave therapy on erectile dysfunction – a double-blinded, sham-controlled, randomized clinical trial. *J Sex Med* 2017;14:106-112. https://doi.10.1016/j.jsxm.2016.11.307.

70. Kalyvianakis D, Hatzichristou D. Low-intensity shockwave therapy improves hemodynamic parameters in patients with vasculogenic erectile dysfunction: a triplex ultrasonography-based sham-controlled trial. *J Sex Med* 2017;14:891-897. https://doi.10.1016/j.jsxm.2017.05.012.

71. Tsai CC, Wang CJ, Lee YC, et al. Low-intensity extracorporeal shockwave therapy can improve erectile function in patients who failed to respond to phosphodiesterase type 5 inhibitors. *Am J Mens Health* 2017;11:1781-1790. https://doi.10.1177/1557988317721643.

72 Bechara A, Casabé A, De Bonis W, Ciciclia PG. Twelve-month efficacy and safety of low-intensity shockwave therapy for erectile dysfunction in patients who do not respond to phosphodiesterase type 5 inhibitors. *Sex Med* 2016;4:e225-e232. https://doi.10.1016/j.esxm.2016.06.001.

73. Frey A, Sønksen J, Fode M. Low-intensity extracorporeal shockwave therapy in the treatment of postprostatectomy erectile dysfunction: a pilot study. *Scand J Urol* 2016;50:123-7. https://doi.10.3109/21681805.2015.1100675.

74. Hisasue S, China T, Horiuchi A, et al. Impact of aging and comorbidity on the efficacy of low-intensity shock wave therapy for erectile dysfunction. *Int J Urol* 2016;23:80-4. https://doi.10.1111/iju.12955.

75. Kitrey ND, Gruenwald I, Appel B, Shechter A, Massarwa O, Vardi Y. Penile low intensity shock wave treatment is able to shift pde5i nonresponders to responders: a double-blind, sham controlled study*. J Urol* 2016;195:1550-1555. https://doi.10.1016/j.juro.2015.12.049.

76. Bechara A, Casabé A, De Bonis W, Nazar J. [Effectiveness of low-intensity extracorporeal shock wave therapy on patients with Erectile Dysfunction (ED) who have failed to respond to PDE5i therapy. A pilot study]. *Arch Esp Urol* 2015;68:152-60. Spanish.

77. Chung E, Cartmill R. Evaluation of clinical efficacy, safety and patient satisfaction rate after low-intensity extracorporeal shockwave therapy for the treatment of male erectile dysfunction: an Australian first open-label single-arm prospective clinical trial. *BJU Int* 2015;115:46-9. https://doi.10.1111/bju.13035.

78. Olsen AB, Persiani M, Boie S, Hanna M, Lund L. Can low-intensity extracorporeal shockwave therapy improve erectile dysfunction? A prospective, randomized, double-blind, placebo-controlled study. *Scand J Urol* 2015;49:329-33. https://doi.10.3109/21681805.2014.984326.

79. Pelayo-Nieto M, Linden-Castro E, Alias-Melgar A, et al. Linear shock wave therapy in the treatment of erectile dysfunction. *Actas Urol Esp* 2015;39:456-9. https://doi.10.1016/j.acuro.2014.09.010.

80. Reisman Y, Hind A, Varaneckas A, Motil I. Initial experience with linear focused shockwave treatment for erectile dysfunction: a 6-month follow-up pilot study. *Int J Impot Res* 2015;27:108-12. https://doi.10.1038/ijir.2014.41.

81. Ruffo A, Capece M, Prezioso D, et al. Safety and efficacy of low intensity shockwave (LISW) treatment in patients with erectile dysfunction. *Int Braz J Urol* 2015;41:967-74. https://doi.10.1590/S1677-5538.IBJU.2014.0386.

82. Srini VS, Reddy RK, Shultz T, Denes B. Low intensity extracorporeal shockwave therapy for erectile dysfunction: a study in an Indian population. *Can J Urol* 2015;22:7614-22.

83. Yee CH, Chan ES, Hou SS, Ng CF. Extracorporeal shockwave therapy in the treatment of erectile dysfunction: a prospective, randomized, double-blinded, placebo controlled study. *Int J Urol* 2014;21:1041-5. https://doi.10.1111/iju.12506.

84. Gruenwald I, Appel B, Vardi Y. Low-intensity extracorporeal shock wave therapy--a novel effective treatment for erectile dysfunction in severe ED patients who respond poorly to PDE5 inhibitor therapy. *J Sex Med* 2012;9:259-64. https://doi.10.1111/j.1743-6109.2011.02498.x.

85. Palmieri A, Imbimbo C, Creta M, Verze P, Fusco F, Mirone V. Tadalafil once daily and extracorporeal shock wave therapy in the management of patients with Peyronie's disease and erectile dysfunction: results from a prospective randomized trial. *Int J Androl* 2012;35:190-5. https://doi.10.1111/j.1365-2605.2011.01226.x.

86. Vardi Y, Appel B, Kilchevsky A, Gruenwald I. Does low intensity extracorporeal shock wave therapy have a physiological effect on erectile function? Short-term results of a randomized, double-blind, sham controlled study. *J Urol* 2012;187:1769-75. https://doi.10.1016/j.juro.2011.12.117.

87. Vardi Y, Appel B, Jacob G, Massarwi O, Gruenwald I. Can low-intensity extracorporeal shockwave therapy improve erectile function? A 6-month follow-up pilot study in patients with organic erectile dysfunction. *Eur Urol* 2010;58:243-8. https://doi.10.1016/j.eururo.2010.04.004.

1. Priapism-induced ED (45 days post-emergency management) [↑](#footnote-ref-1)
2. ED on the basis of chronic pelvic pain syndrome [↑](#footnote-ref-2)
3. Veno-occlusive ED based on hypogonadotropic hypogonadism [↑](#footnote-ref-3)
4. No IIEF-EF score at any follow-up time (M1, M6, M12, M18) reported [↑](#footnote-ref-4)
5. post pelvic fractures associated with urethral injury [↑](#footnote-ref-5)
6. ED in kidney transplant recipients (multifactorial) [↑](#footnote-ref-6)
7. Only data of "LI-ESWT responders" reported (24 of 40 analyzed patients, with 50 patients included in the study and 10 patients lost to follow-up) [↑](#footnote-ref-7)
8. Netherlands, Palestine, Lithuania and Czech Republic [↑](#footnote-ref-8)
9. This is a retrospective analysis of patients who were treated with the same LI-ESWT protocol, with essential information (device used not specified). The authors divided their sample into two subsamples according to different IIEF-EF scores at baseline. Here this study is considered a case series without control group. [↑](#footnote-ref-9)
10. Shenzhen Hyde Medical Equipment [↑](#footnote-ref-10)
11. Intellect Focus Shockwave Therapy SKU [↑](#footnote-ref-11)
12. Zhanjiang Haibin Medical Equipment Co [↑](#footnote-ref-12)
13. Mean [↑](#footnote-ref-13)
14. Median [↑](#footnote-ref-14)
15. Standard deviation [↑](#footnote-ref-15)
16. Range [↑](#footnote-ref-16)
17. Minimum value or range [↑](#footnote-ref-17)
18. Approximately 25% of the patients were lost to follow-up. However, group-specific patients lost to follow-up were not reported. [↑](#footnote-ref-18)
19. Approximately 40% of the patients were lost to follow-up. However, group-specific patients lost to follow-up were not reported. [↑](#footnote-ref-19)
20. Energy that is used to electromagnetically accelerate the bullet in certain radial ESWT devices (c.f. Figure 2D in the main text). [↑](#footnote-ref-20)
21. Air pressure that is used to accelerate the bullet in pneumatic, radial ESWT devices (c.f. Figure 2D in the main text). [↑](#footnote-ref-21)
22. Current that is rapidly discharged across two electrode tips (spark-gap) to vaporize the surrounding water in electrohydraulic ESWT devices (c.f. Figure 2A in the main text). [↑](#footnote-ref-22)
23. 14 patients treated with 12 treatment sessions and 5 patients treated with 6 treatment session; all patients were combined to a single group. [↑](#footnote-ref-23)
24. 20 mJ/mm^2^, 15 mJ/mm^2^ and 12mJ/mm^2^ (these values are most probably incorrect; details in the Discussion section of the main text. [↑](#footnote-ref-24)
25. Non-responders received a second series of 6 treatment sessions (W7-W9 (2)); all patients were combined to a single group. [↑](#footnote-ref-25)
26. Mean [↑](#footnote-ref-26)
27. Median [↑](#footnote-ref-27)
28. Standard deviation [↑](#footnote-ref-28)
29. Range [↑](#footnote-ref-29)
30. Minimum value or range [↑](#footnote-ref-30)
31. Approximately 25% of the patients were lost to follow-up. However, group-specific patients lost to follow-up were not reported. [↑](#footnote-ref-31)
32. Approximately 40% of the patients were lost to follow-up. However, group-specific patients lost to follow-up were not reported. [↑](#footnote-ref-32)
33. Mean LLEF-EF score at baseline [↑](#footnote-ref-33)
34. Standard deviation of the LLEF-EF score at baseline [↑](#footnote-ref-34)
35. Mean LLEF-EF score at follow-up [↑](#footnote-ref-35)
36. Standard deviation of the LLEF-EF score at follow-up [↑](#footnote-ref-36)
37. Range [↑](#footnote-ref-37)
38. 95% confidence interval of the mean [↑](#footnote-ref-38)
39. The authors stated that they performed a modified ITT analysis. To this end, patients who were found ineligible after randomization were excluded (i.e. patients who met all inclusion criteria but with a baseline IIEF-EF score > 25). However, an IIEF-EF score ≥ 25 was an exclusion criteria used in this trial, raising the question why patients were randomized into the trial despite the fact that they fulfilled one of the exclusion criteria and were therefore excluded after randomization. Furthermore, patients with missing primary outcome data were also excluded. However, this should be considered lack of missing data imputation rather than performing a modified ITT analysis. [↑](#footnote-ref-39)
40. Patients with de novo use of erectogenic aids at the first follow-up were excluded from the analysis. [↑](#footnote-ref-40)
